# Lens-free microscopy for 3D + time acquisitions of 3D cell culture

**DOI:** 10.1038/s41598-018-34253-6

**Published:** 2018-10-31

**Authors:** Anthony Berdeu, Bastien Laperrousaz, Thomas Bordy, Ondrej Mandula, Sophie Morales, Xavier Gidrol, Nathalie Picollet-D’hahan, Cédric Allier

**Affiliations:** 1 Université Grenoble Alpes, Grenoble, F-38000 France; 2 Commissariat à l’énergie atomique et aux énergies alternatives, Laboratoire d’électronique et de technologie de l’information, Grenoble, F-38054 France; 3 Commissariat à l’énergie atomique et aux énergies alternatives, Biologie à Grande Echelle, Grenoble, F-38054 France; 4 Institut national de la santé et de la recherche médicale, U1038, Grenoble, F-38054 France

## Abstract

Thanks to a novel three-dimensional imaging platform based on lens-free microscopy, it is possible to perform multi-angle acquisitions and holographic reconstructions of 3D cell cultures directly into the incubator. Being able of reconstructing volumes as large as ~5 mm^3^ over a period of time covering several days, allows us to observe a broad range of migration strategies only present in 3D environment, whether it is single cell migration, collective migrations of cells and dispersal of cells. In addition we are able to distinguish new interesting phenomena, e.g. large-scale cell-to-matrix interactions (>1 mm), fusion of cell clusters into large aggregate (~10,000 µm^2^) and conversely, total dissociation of cell clusters into clumps of migrating cells. This work on a novel 3D + time lens-free microscopy technique thus expands the repertoire of phenomena that can be studied within 3D cell cultures.

## Introduction

Recently the imaging of 3D cell cultures opened a new window onto the study of many cellular processes as nicely reviewed in^1^. 3D + time imaging of 3D cell culture is usually performed through optical sectioning microscopy techniques, e.g. light-sheet microscopy and confocal live-cell microscopy. Light-sheet microscopy is ideally suited to monitor 3D cell culture, it can acquire large volume in reasonable time and with minimal photo-toxicity. However, it requires the sample to be labelled with fluorescent dyes and the geometry of the sample container is constrained. This is not yet the ultimate gentle microscope as defined in^2^, that is needed for the future experimentations. A gentle microscope should be adapted to the sample, without any modification of its environment nor its integrity. In particular it should be compatible with all kind of cell culture container and if possible label-free.

With the aim of developing such a gentle microscope, we developed a novel 3D + time lens-free microscope dedicated to the observation of dynamic biological processes present in 3D cell culture as previously presented in^3^. It is based on the 3D lens-free microscopy setup introduced in^4^ which enables a large angular coverage of the 3D scene thanks to its azimuthal acquisition geometry. This setup was modified to perform continuous monitoring inside an incubator at a controlled temperature and humidity^3^. The temperature of the CMOS sensor facing the 3D cell culture is now controlled by means of a laminar air flow which enables to run the image sensor without heating up the cell culture. This allows for the first time 3D + time lens-free acquisitions of 3D cell culture. This microscope works thus directly in the incubator with a regular cell culture container and is able to reconstruct large volumes of label-free 3D cell culture (~5.6 mm^3^).

The present paper follows our previous work^3^, which introduced the experimental design to perform 3D + time lens-free acquisitions of 3D cell culture. Here we demonstrate the ability of this novel setup to gain insights into a broad range of phenomena only present in 3D environments. We discuss the analysis of two experiments of 3D cell culture of RWPE-1 cells acquired over eight consecutive days. RWPE-1 cells are a model for normal prostate epithelial cell behavior characterized by a polarized acinar morphology in 3D cultures^5,6^. RWPE-1 cells have also been used as a dynamic model of the signaling and interactions between organoids and mesenchyme that are required during organ development^7^. Observing volumes as large as 5.6 mm^3^ over several days allows the visualization of a broad range of cell migration patterns discussed in^8,9^, such as the migration of cell leaders, collective cell migration and close-gap branching. We also witnessed interesting new phenomena, such as the cohesive migration of large aggregates of cells, the growth of cell clusters through the aggregation of isolated cells and conversely, the dissociation of cell clusters into clumps of single cells. In addition, we successfully monitored the dynamic evolution of the extracellular matrix on a global scale and we were able to isolate the matrix deformations resulting from traction forces generated by large cell aggregates over long distances, up to 1.5 mm. All these observations demonstrate that many important features of cell migration and cells-ECM (extra cellular matrix) interactions can be conveniently observed with our novel 3D + time lens-free microscope.

## Methods

### Cell culture

The RWPE-1 cell line was obtained from ATCC (CRL-11609). This cell line is derived from non-neoplastic human prostate epithelial cells by immortalization with human papillomavirus. RWPE-1 cells were maintained in KSFM (Life Technologies) supplemented with 5 ng/mL Epidermal Growth Factor (Life Technologies), 50 mg/mL Bovine Pituitary Extract (Life Technologies) and 1% Penicillin-Streptomycin (Life Technologies). Cells were passaged upon 70% confluence and seeded at 20000 cells/ml density. The cells were routinely cultured in a humidified atmosphere with 5% CO_2_ at 37 °C. For the 3D cell culture experiments a Matrigel® drop was deposited at the center of Greiner petri dishes (Sigma-Aldrich) and allowed polymerizing for 30 minutes at 37 °C. RWPE-1 cells were then added at the surface of Matrigel and allowed attaching for 1 hour at 37 °C. KSFM (Life Technologies) supplemented with 50 ng/mL Epidermal Growth Factor (Life Technologies), 2% Fetal Bovine Serum (Life Technologies) and 1% Penicillin-Streptomycin (Life Technologies) was subsequently added and cell culture was monitored by 3D lens-free imaging for 7 days. For the second experiment, fluorescent beads with a diameter of 10 µm (Sigma-Aldrich) were mixed with Matrigel, prior to cell seeding.

### Experimental setup

**Figure 1:**
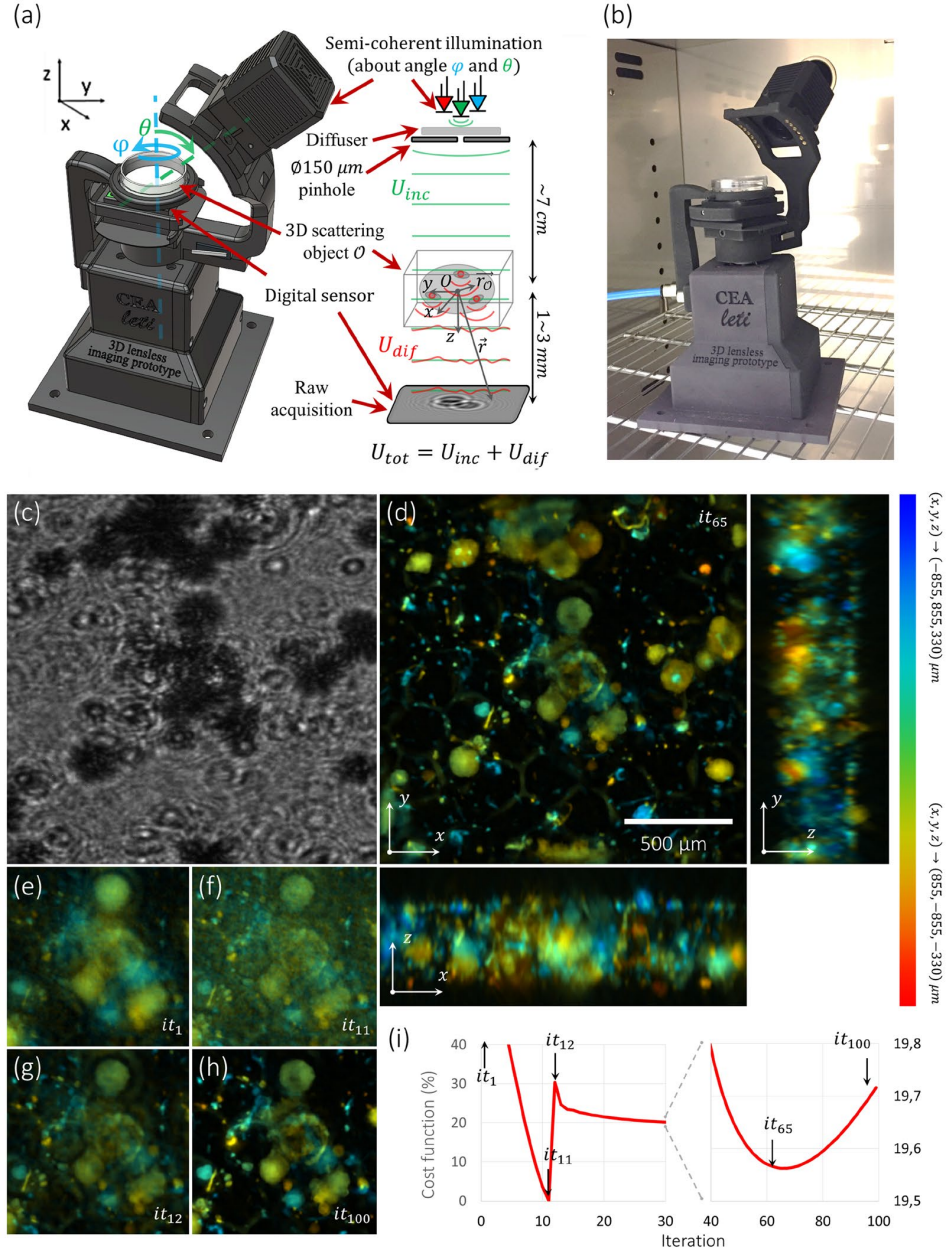
Experimental setup dedicated to lens-free diffractive tomography and reconstruction of a culture of RWPE-1 cell embedded in Matrigel capsules^3^. (**a**) Optical scheme of the system: LEDs placed behind a pinhole create a semi-coherent incident plane wave *U*_*inc*_ which is scattered by the 3D sample. This creates a diffracted wave *U*_*dif*_. These waves interfere on the sensor which records the resulting intensity: ${I}_{d}={|{U}_{inc}+{U}_{dif}|}^{2}$. (**b**) Picture of the experimental setup installed into the cell culture incubator. The Petri dishes are 35 mm in diameter. (**c**) Lens-free raw data acquisition of a 3D cell culture of RWPE-1 cells (cropped image from a dataset of 3 × 31 acquisitions in *RGB* with 31 angles in $\phi \in \{0^\circ ,282^\circ \}$ with ${\rm{\Delta }}\phi =9.4^\circ ,\theta =45^\circ$). (**d**) 3D orthogonal average intensity projection of the reconstructed volume obtained by the regularized Gerchberg-Saxton algorithm at the iteration *it* = 65 on a 3D volume of 1.7 × 1.7 × 1.6 = 4.7 mm^3^, 512 × 512 × 300 voxels of 3.34 × 3.34 × 3.34 = 3703 μm^3^. The objects have been color-coded to visualize their position along the different axis of projection. The color gives the depth in the volume for each view. The blue color encodes for the highest positions (*z* = 330 µm for the *xy*-view, *x* = −855 µm for the *yz*-view, *y* = 855 µm for the *xz*-view) and the red color encodes for the deepest positions (*z* = −330 µm for the *xy*-view, *x* = 855 µm for the *yz*-view, *y* = −855 µm for the *xz*-view). (**e**–**h**) *xz*-average intensity projections at the center of the reconstructed volume at different iterations *it* ∈ {1, 11, 12, 100}. (**i**) Evolution of the data fidelity term in the cost function during the iterations normalized to its minimum and maximum values. Black arrows point at the iterations shown on (**d**–**h**).

3D objects reconstruction requires to multiply the viewing angles. In our experimental bench (see Fig. 1a), the CMOS sensor (29.4 mm^2^, 3840 × 2748 monochromatic pixels, pixel pitch 1.67 μm - ref. UI-1942LE-M) and the multi-wavelength illumination source (LED Cree, ref. XLamp MC-E RGBW MCE4CT-A2, *λ*_*B*_ = 450 nm; *λ*_*G*_ = 520 nm; *λ*_*R*_ = 640 nm) are rotated by a stepper motor (ref. RS-PRO-535-0401). The object remains static, while the rotation axis is orthogonal to the sample plane. The illumination is tilted by an angle *θ*, tuned between 30 and 55°. As the sensor is kept in the same plane, this design is adapted to extended containers such as standard Petri dishes. An air knife blown via a vein carved in the sample holder insures the thermal insulation of the cell culture from the heat produced by the CMOS censor (see Fig. S1a).

### Holographic 3D reconstruction

The purpose of the holographic reconstruction algorithm is to recover the 3D scattering potential of the sample from datasets consisting of holographic images recorded at different illumination positions and at different wavelengths. Figure 1d shows an example of 3D holographic reconstruction. For the fully 3D reconstruction, we used two different algorithms based on the Fourier diffraction theorem and the Born approximation of the light propagation. The first 3D reconstruction algorithm is based on the 3D inverse problem approach (see supplementary informations). It gives the best results in terms of contrast and quality^4^. It is, however, time consuming and is used only to reconstruct small region of interests at full resolution (voxel of 1.67 × 1.67 × 1.67 = 4.7 μm^3^). The second 3D reconstruction algorithm is based on a regularized Gerchberg-Saxton algorithm introduced in^3^ in which constraints are applied on the 3D objects^10,11^ (see supplementary informations). For the fast reconstruction of volume as large as ~5 mm^3^, we used the regularized Gerchberg-Saxton algorithm at a lower resolution (voxel of 3.34 × 3.34 × 5.32 = 60 μm^3^), providing sufficient quality results.

The assessment of the 3D holographic reconstruction algorithms in terms of position accuracy and capabilities of size estimation has been performed using a reference object made of 10 μm beads embedded into Matrigel. We compared the 3D reconstruction obtained from the lens-free acquisitions with the Z-stack acquisitions obtained with a fluorescence microscope (Zeiss axio Observer Z1, 5x/0.13 N.A.). The 3D lens-free microscope operating with an illumination angles of *θ* = 45° achieves good position accuracy. We measured on *N* = 409 micro-beads an overall localization error of 3.8 μm (standard deviation) on the xy plane and 6.4 μm along the *z*-axis (Supplementary Table 1). Considering the size estimation measured on the 10 μm beads, the full width at half maximum (FWHM) value measured for the *xy* line profile of 10 μm beads is estimated to 10.5 μm (Supplementary Table 2). But along the z-axis the FWHM is in the order of 50 μm (Supplementary Table 2). The sizing is thus correct in the *xy* plane but it is strongly overestimated along the *z*-axis. The performance of the lens-free 3D microscope in terms of position accuracy and *xyz*-sizing are therefore relatively coarse but sufficient to analyze the dimensions of the cell clusters a relative manner.

## Results

### First experiment of 3D time-lapse acquisition

#### The merging of cell clusters into large aggregate

**Figure 2:**
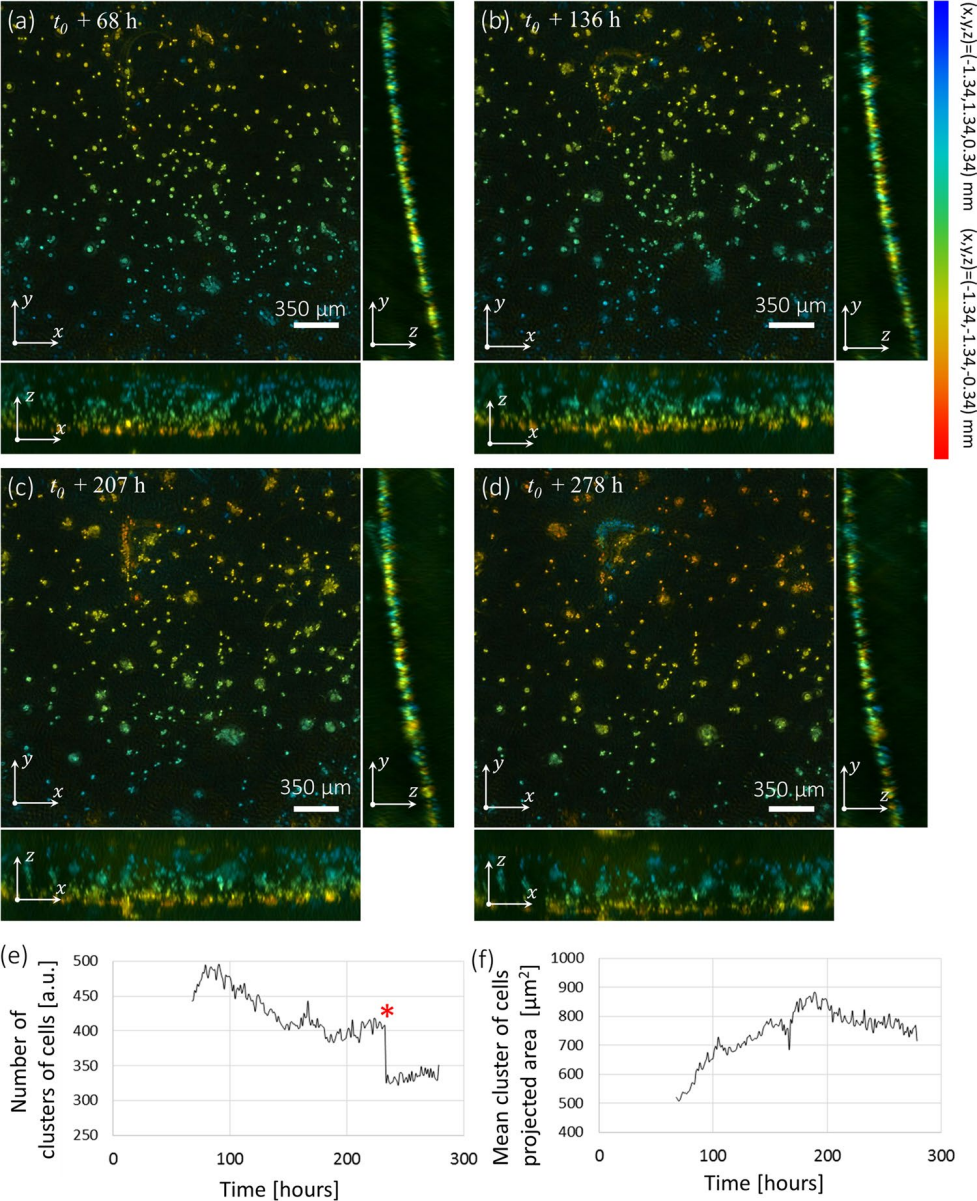
First experiment of 3D + time lens-free microscopy^3^. (**a**–**d**) 3D orthogonal average intensity projection of the reconstructed volume of a 3D culture of RWPE-1 cells obtained with the 3D lens-free microscope. The four 3D reconstructions are taken from a 210 h long time-lapse acquisition at respectively *t*_0_ + 68 h, *t*_0_ + 136 h, *t*_0_ + 207 h and *t*_0_ + 278 h, *t*_0_ being the initial time of the cell culture experiment. The regularized Gerchberg-Saxton algorithm was run with the following parameters: $\phi \in \{0^\circ ,305^\circ \}$, $\Delta \phi =9.8^\circ ,\theta =45^\circ$, 3D volume = 2.67 × 2.67 × 0.68 = 4.86 mm^3^, 800 × 800 × 128 voxels of 3.34 × 3.34 × 5.32 = 60 μm^3^. The clusters of cells have been color-coded to visualize their position along the different axis of projection. The color gives the depth in the volume for each view. The blue color encodes for the highest positions (*z* = 0.34 mm for the *xy*-view, *x* = −1.34 mm for the *yz*-view, *y* = 1.34 mm for the *xz*-view) and the red color encodes for the deepest positions (*z* = −0.34 mm for the *xy*-view, *x* = 1.34 mm for the *yz*-view, *y* = −1.34 mm for the *xz*-view). The red and green regions of interest emphasize noticeable cell behaviors (see Fig. 3d–g). (**c**) Maximum intensity projection of the temporal stack for each pixel of the *xy*-view. (**e**) Number of detected objects as a function of the experiment time. The red star indicates the change of culture media at *t*_0_ = 234 h when several clusters of cells have detached. (**f**) Mean clusters of cells projected area as a function of time.

Figure 2 shows the 3D time-lapse acquisition obtained with our lens-free microscope on a 3D cell culture of RWPE-1 (see Movie S1). The dimensions of the 3D reconstructed volume obtained by the regularized Gerchberg Saxton algorithm is 4.86 mm^3^ (2.67 mm × 2.67 mm × 0.68 mm) and it is hence possible to monitor hundreds of clusters of cells objects simultaneously (*N* > 300 Fig. 2c).

**Figure 3:**
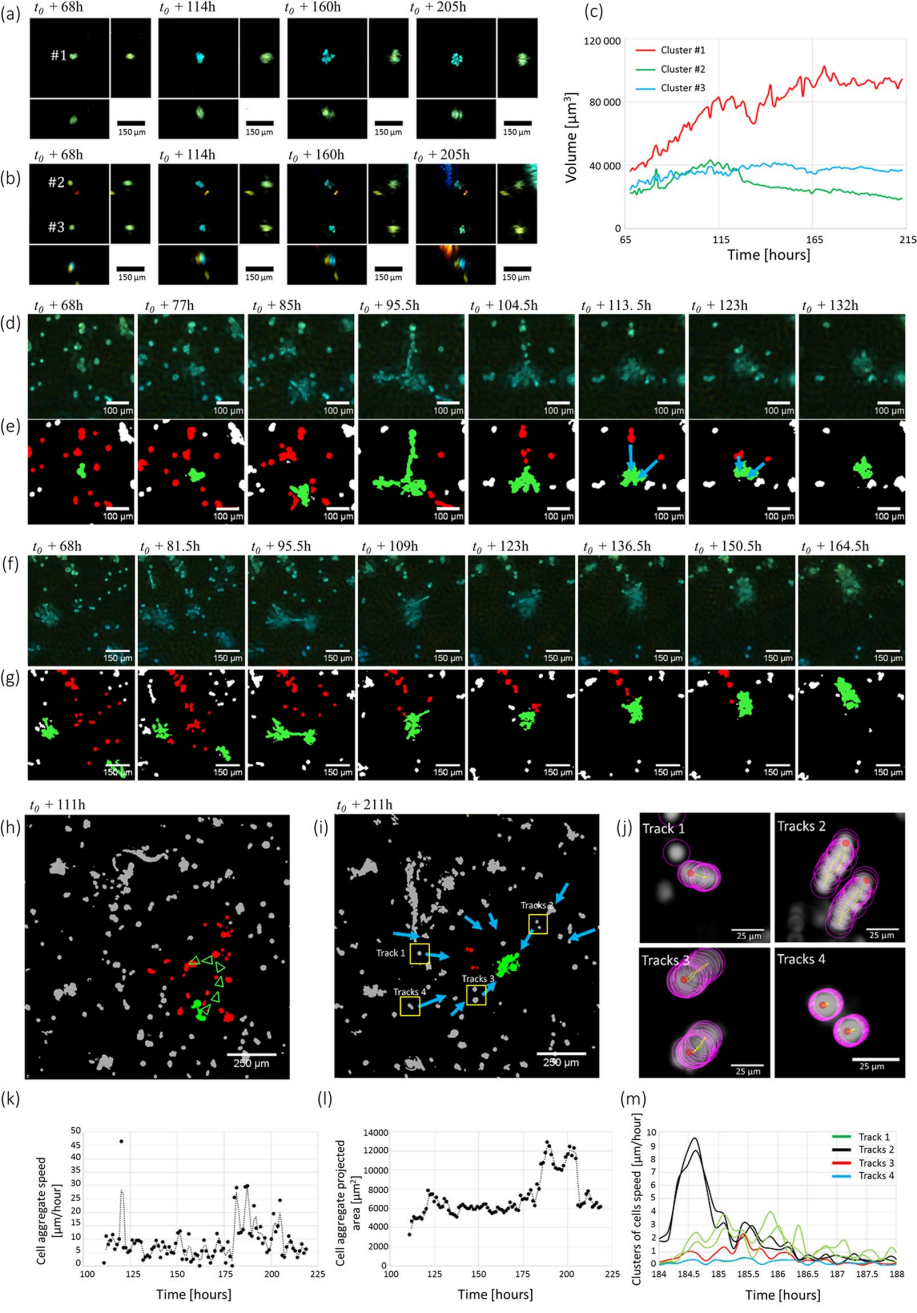
Different phenomena of cell cluster growth. (**a**,**b**) Time series of the reconstructed volume of three clusters of cells. The color gives the depth as in Fig. 2a. (**c**) Plot of the volume of the cells clusters as a function of time. The volume is computed with a simple thresholding. (**d–i**) Time series of region of interest highlighted in red in Fig. 2a showing the merging of cells into large aggregates. The corresponding time-lapse acquisitions are further shown in Movies S2 and S3. (**e**) and (**g**) The *xy*-projections depicted in respectively (**d**) and (**f**) are segmented and a color code is given to the different clusters of cells, *i.e*. green for the cluster of interest and red for the cells that will be aggregated into this cluster. Other cells that were not aggregated to this cluster are depicted in gray. In (**e**) the blue arrows are pointing to the small cell clusters attracted to the large aggregate (green). (**h,i**) *xy*-snapshots at *t*_0_ + 111h and *t*_0_ + 211h of a region of interest highlighted in green in Fig. 2a and reconstructed at full resolution with the inverse approach algorithm. The color code the depth as in Fig. 2a. In (**h**), the green arrows show the displacement of the large aggregate. In (**i**), the blue arrows point at cells fixed in the Matrigel which highlight its deformation as they move towards the large aggregate. (**j**) Visualizations of cell displacements resulting from the traction forces created by the large aggregate onto the Matrigel. The initial positions of the clusters are shown in (**i**). The cell tracking has been performed with the Fiji plugin Trackmate^23^. The cluster detection is shown with purple circles, the track in yellow and the starting point in red. (**k**) Speed of the large aggregate shown in green in (**h**) as a function of time. (**l**) Total projected area of the large aggregate shown in green in (**h**) as a function of time. (**m**) Speed of the cells clusters depicted in (**i,j**) as a function of time.

Analyzing the dimensions of the cell clusters present in the time-lapse acquisition, we found that the average *xy* projected area increased from 500 to 710 μm^2^ in about 200 hours (see Fig. 2d). A first population of isolated clusters grew via cell proliferation like the one depicted in Fig. 3a but interestingly other clusters increased in size through merging. As a first example, Fig. 3d,e and Movie S3 depict a cluster which grew by attracting to itself 15 other clusters. At *t*_0_ + 95 h, this cluster generated three extensions of 70 to 200 μm in length in order to attract a large number of cells. At *t*_0_ + 185 h, it became unstable. Three times it ejected small clusters of cells, in the direction of another cell aggregate located at a distance of 300 µm (see Movie S3). Figure 3f,g and Movie S4 show another interesting phenomena, *i.e*. the merging of two cell aggregates consecutive to apparent pairwise attraction. At *t*_0_ + 68h, the two aggregates are about 55 μm in diameter and they are separated by a large distance of 450 μm. They first moved towards each other, accumulating cells from the surroundings. Before the final merging, branches emerged and connected the two aggregates. The aggregate continued to move a little, by 150 μm and accumulated more neighboring cells. This process of cell dynamic clustering is strikingly depicted in Fig. 3h,i and Movie S5 which shows the another accumulation of 25 clusters into one very large aggregate of ~6000 μm^2^. The newly formed aggregate still managed to move as a cohesive group at a speed of 5–10 µm/h. Its course presents speed maxima (~30 μm/h, Fig. 3k) which corresponds to sequence of expansion and contraction. At *t*_0_ + 184h, following a large expansion, we observed a traction force generated by the aggregate onto the ECM. This could be indirectly observed through the displacement of several clusters towards the large aggregate (Fig. 3j). These tractions forces disappeared as the aggregate released the tensions applied to the ECM and the clusters moved back to their initial positions. The traction forces were generated isotropically and they could be observed up to a distance of 550 μm. A similar observation is shown in Fig. 3e and Movie S3 at *t*_0_ + 120h but at a smaller scale.

### Second experiment of 3D time-lapse acquisition

#### The formation of a complex cellular network

**Figure 4:**
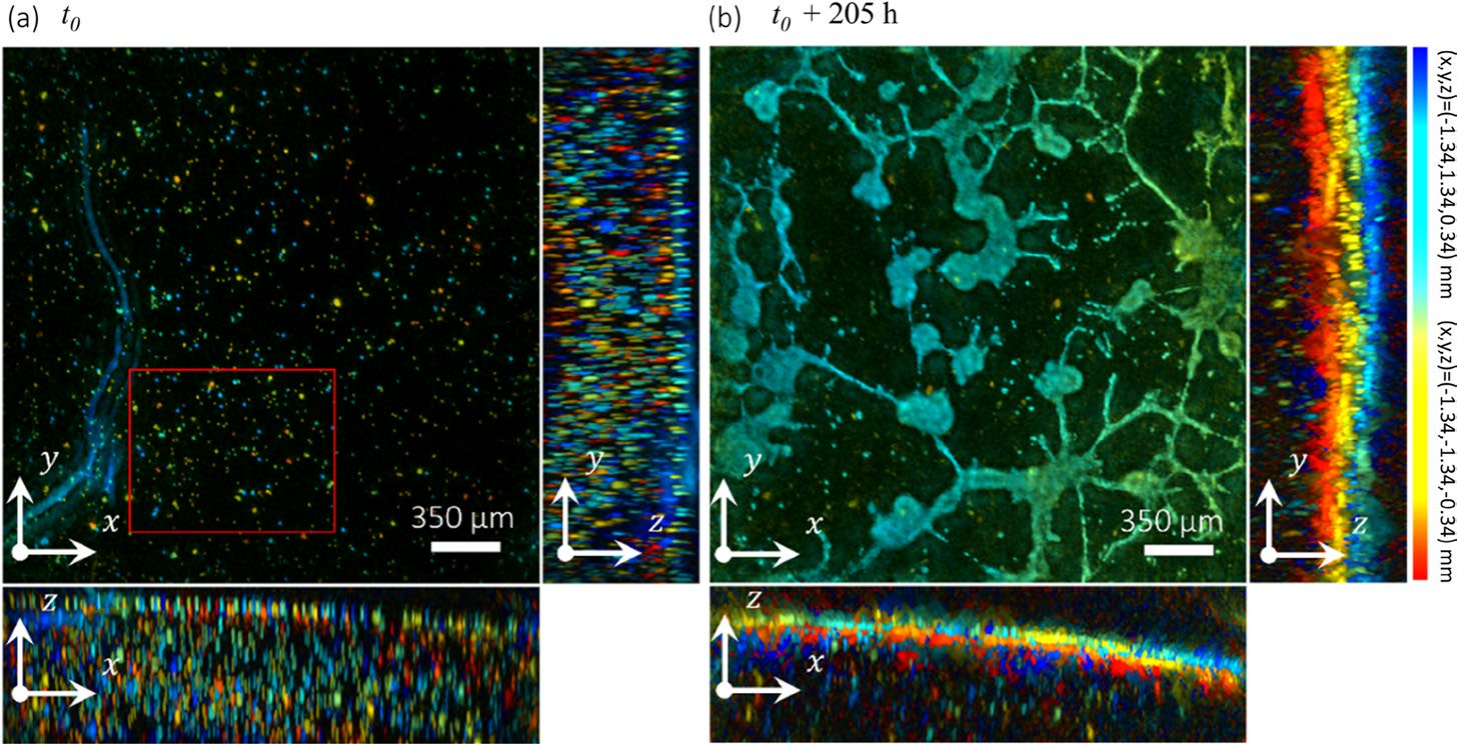
Second experiment of 3D + time lens-free microscopy. (**a**,**b**) 3D orthogonal average intensity projection of the reconstructed volume of a 3D culture of RWPE-1 cells with 10 μm beads embedded into the ECM. The *xz*-projections show clearly the presence of the 10 μm beads into the ECM. The two 3D reconstructions are taken from a 8 days h long time-lapse acquisition at the start end the end respectively. The views are (*xy*) and (*xz*) intensity projections of the reconstructed volumes. The regularized Gerchberg-Saxton algorithm was run with the following parameters: $\phi \in \{0^\circ ,305^\circ \}$, $\Delta \phi =9.8^\circ ,\theta =45^\circ$, 3D volume = 2.67 × 2.67 × 0.78 = 5.57 mm^3^, 800 × 800 × 128 voxels of 3.34 × 3.34 × 6.10 = 68 μm^3^. The clusters of cells have been color-coded to visualize their position along the different axis of projection. The color gives the depth in the volume for each view. The blue color encodes for the highest positions (*z* = 0.37 mm for the *xy*-view, *x* = −1.34 mm for the *yz*-view, *y* = 1.34 mm for the *xz*-view) and the red color encodes for the deepest positions (*z* = −0.37 mm for the *xy*-view, *x* = 1.34 mm for the *yz*-view, *y* = −1.34 mm for the *xz*-view). The red square is the region of interest presented in Fig. 5.

**Figure 5:**
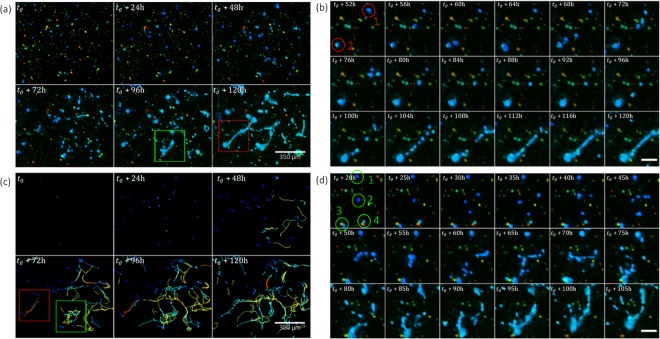
Formation of cell branches initiated by dissociations of cell clusters and single cell migrations. (**a**) Time series of a region of interest (red rectangle in Fig. 4a) showing the formation of a large cellular structure. (**c**) Cell tracking performed on the time-lapse shown in (**a**), obtained with the Fiji plugin Trackmate^23^. Each cell trajectories are 24 hours long. The color code gives the speed, the warmer, the faster (30 µm/h). The red and green rectangles point branches that are visible in the cell tracking time-series before the effective formation of cell branching. The latter are pointed by rectangles in (**a**), latter on in the time series. (**b**) Time series of a region of interest, a detail of (**a**), showing the formation of a cell branching. The latter is initiated by the dissociation of cluster 1 into two clusters, one fixed and one moving towards cluster 2. In the following hours a large number of migrating cells follows the exact same path between clusters 1 and 2, which, ultimately form a branch between the two clusters. (**d**) Time series of a region of interest, a detail of (**a**), showing the formation of a cell branching. In this region, there are initially four cell clusters. Two of them (clusters 2 and 4) completely dissociated into clumps of migrating cells, while the two others (clusters 2 and 3) remained fixed and continued to grow. A lot of cell trafficking occurred in between the two fixed clusters and ultimately a cell branching connected the two clusters.

In contrast to the first experiment where we observed cell clusters moving, deforming and merging, in this second experiment we observed the construction of a complex and large cellular network, *i.e*. a long-scale tubular pattern (see Fig. 4 and Movie S6). In the first hours of the cell culture, we observed the dissociation of cell clusters into clumps of single migrating cells. As a first example, Fig. 5b and Movie S7 show the dissociation at *t*_0_ + 53h of a cell cluster into a fixed cluster and a migrating cluster. The latter moved along a linear path directly toward another fixed cluster at a distance of 450 μm.

This path is taken by several cells, migrating between the two fixed clusters in both directions. At *t*_0_ + 120h, the two fixed clusters are finally connected by a branch of 200 μm in length. A second example, in Fig. 5d and Movie S8, depicts the total dissociation of a cluster into four single cells. These cells further migrated along linear paths, they followed each other, sometimes turning back. Importantly they formed trains of cells groups and in a second step, several paths taken by these cells became gradually branches of cells connecting two fixed clusters initially separated by ~300 μm. This phenomenon of dynamic branches formation is best observed when comparing the formation of the cellular network (Fig. 5a) with the trajectories of the single cells (Fig. 5c). In the cell trajectories time-series, one can observe branches of 100 to 500 μm in length well before the effective formation of the cell branching that occurred 24 to 48 hours later. This phenomenon of dynamic branching happened all over the cell culture and after six days a complex cellular network was formed featuring ~30 aggregates in the range of 50 to 100 μm in diameter (see Fig. 4b) interconnected with branches of 100 to 500 μm in length.

Finally, after 6 days of experiment, the cellular network collapsed as branches of cells and cell aggregates merged together (see Movie S6). Yet, the cellular network did not vanished completely. In sum, we can distinguish three distinct phases in this RWPE-1 3D cell culture experiment that led to a complex cellular network, *i.e*. a first phase of single cell migration (~96 h), a second phase of interconnection between cell aggregates (~48 h), and a final phase where the cellular network collapsed partially (~60 h).

#### The generation of long-scale traction forces

**Figure 6:**
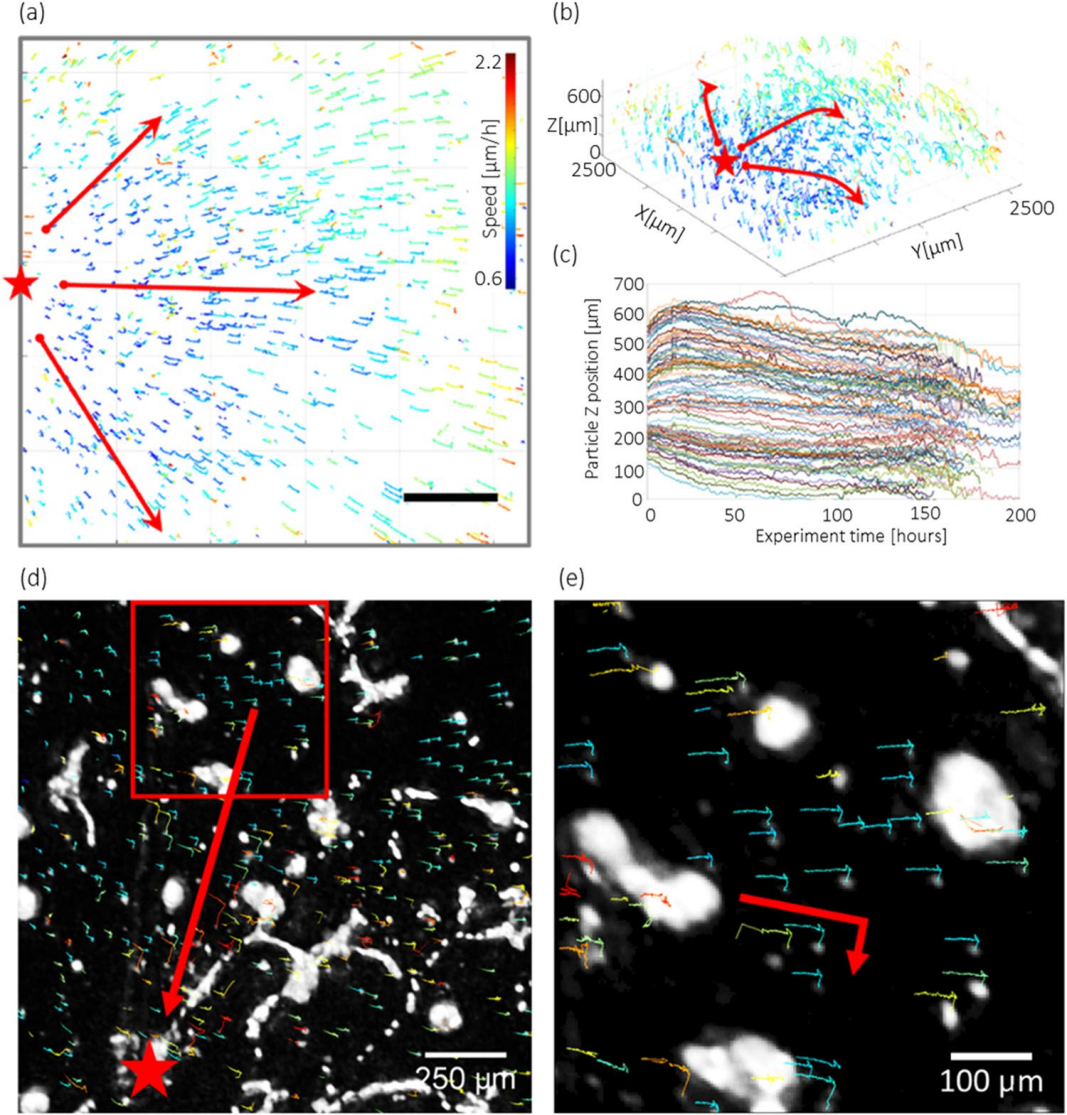
Deformation of the ECM and long-range traction forces. (**a**,**b**) 3D particle tracking of the beads in the *xy*-projection (**a**) and the 3D volume (**b**). Tracking was performed with the trackmate Fiji plug-in^23^ using the *xy*-projection. The figure shows the tracks of the 10 μm beads over 208 hours, with a color code corresponding to their median speed. The red arrows show the overall direction of the tracks. The red star shows the center of the ECM radial dilation. (**c**) Plot of the tracks of 250 beads along the *z*-direction as a function of time. (**d**) Snapshot of a region of interest at *t*_0_ + 136 h showing a long-scale deformation of the ECM consecutive to traction forces generated by the merging of several cell clusters (red star) slightly deeper below the Matrigel surface (see Supplementary Movie S9). The direction of the traction forces is shown with a red arrow. The tracks of the beads are 100 hours long, the color code is the same as in (**a**). (**e**) Details of (**d**) (red box) showing the displacement of the beads towards the cell aggregate at distances ranging between 1000 and 1400 µm. The red arrows highlight the change in direction of the 10 μm beads tracks consecutive to the generation of traction forces onto the ECM.

In order to better visualize the 3D deformations of ECM present in this second experiment, we inserted 10 µm beads at different depths into the ECM prior to the 3D cell culture. This method is usually performed at the microscopic scale to monitor and quantify the single cell traction forces^12^. Here it was used to show the long scale deformations of the ECM. In the first hours of the experiment, the micro-beads allowed us to observe the intrinsic 3D deformations of the ECM. In the *xy*-plan, the ECM expanded radially. All the beads moved away from a central point at a speed increasing with the distance to this point (see Fig. 6a). The speed of the beads was about 1 μm/h close to the center of expansion and about 2 μm/h at a distance of approximately 2.5 mm (see Fig. 6a). At *t*_0_ + 90 h, the expansion stopped and the ECM remained stable in the *xy*-projection. In the *z*-direction, during the first 24 hours, there was an important ECM expansion of about 100 µm followed by a slow drift which lasted until the end of the experiment (see Fig. 6c). At *t*_0_ + 136h, the collapses of the network revealed significant traction forces that distorted the ECM. We isolated a strong ECM deformations resulting from traction forces generated by the collapse of several cell clusters (see Fig. 6d,e and Movie S9). This traction forces moved the beads by 100 μm (speed of 12 μm/h) at a distance of 350 µm from the cell aggregate. Bead displacement can also be observed 70 μm deep into the matrigel (see Movie S9) and at a large distance of 1400 μm (see Fig. 6e). There is thus an important remodeling of the ECM that occurred during the collapse of the cellular network.

## Discussion

In the present paper, we demonstrated the capability of lens-free 3D microscopy to perform 3D + time acquisitions of 3D cell culture. Acquiring a volume as large as ~5.6 mm^3^ over several days, allowed us to observe the broad spectrum of migration mechanisms previously discussed in^8,9,13,14^, whether it is single cell migration, collective migrations of cells, and cell branching. In addition, we observed the cohesive migration of a large aggregate of cells (~10,000 µm^2^ projected area) that confirms the prediction that an isolated cells monolayer may acquire a global polarity and consequently performs a persistent random walk^15^. We also observed the sprouting of cells, i.e. short-range dispersal of cells. This mechanism described in a model discussed in^16^ supports a faster rate of tumor growth.

We have also observed the formation of a complex cellular network of several mm^3^, *i.e*. a long-scale tubular pattern made of large cell aggregates connected by cell branches and surrounded by voids. This spongy structure has its origins in the cell migrations patterns that occurred during the early days of the cell culture. The paths taken by the migrating cells have indeed settled the interconnection between the different aggregates. In particular, we observed total dissociations of cell clusters into clumps of migrating single cells that provide simultaneously a path and the cell material to form new branches. This is a novel process which, is geometrically distinct from the “budding” and “clefting”, the two processes that usually describe branching morphogenesis^17^. This can also be viewed as a new invasive phenotype, the study of which should lead to a better understanding of tumor initiation and metastasis.

Finally, we also studied the deformations of the ECM. We first observed intrinsic deformations of the ECM. The speed of these deformations are in the range of 1 to 2 µm/h. This is not negligible and it should be taken into account when measuring the speed of single cells in 3D cell culture. We were also able to isolate ECM deformations resulting from traction forces generated by cell aggregates. Importantly, we observed ECM deformations at two different scales, namely local ECM deformations generated by an aggregate which favor close-gap branching and large scale deformations that occurred during the collapse of the cellular network. The latter generated traction forces over long distances, up to 1500 µm. To our knowledge, this is the longest reported ECM deformation. Long-range traction forces have already been discussed but for smaller lengths of ~600 µm in^18,19^. 3D + time lens-free microscopy is thus a unique mean to capture in time the dynamic and multi-scale interplay between complex cellular self-assembly and ECM remodeling at different scales. These are important observations, as the dysregulation affecting ECM may contribute to pathologies such as inflammation^8^, age-related diseases^20^ or cancer^21,22^.

## Conclusion

In this paper we demonstrate that the novel 3D + time lens-free microscopy is a unique and powerful technique providing insights into spatial and temporal aspects of 3D cell cultures. Compared to other 3D microscopy techniques, such as confocal microscopy, 3D digital holographic microscopy, and light-sheet microscopy which provide much better resolution and specificity due to fluorescence labelling, our 3D lens-free microscopy technique favours ease of use, label-free experimentation and the acquisition of large field of view 3D + time datasets. To our knowledge, our technique is the only one able to reconstruct large volumes of 3D cell cultures (~5.6 mm^3^) by phase contrast imaging.

## Electronic supplementary material

S1

S2

S3

S4

S5

S6

S7

S8

S9

Supplementary informations
