# Supplementary material for: Lens-free microscopy for 3D + time acquisitions of 3D cell culture
Source: Sci Rep. 2018 Oct 31;8:16135. doi: 10.1038/s41598-018-34253-6 (PMC6208343; doi:10.1038/s41598-018-34253-6)
Supplement: Supplementary file 10 — Supplementary informations [file 41598_2018_34253_MOESM10_ESM.docx]

**Supplementary informations**

Lens-free microscopy for 3D+time acquisitions of 3D cell culture

Anthony Berdeu^1,2^, Bastien Laperrousaz^1,3,4^, Thomas Bordy^1,2^, Ondrej Mandula^1,2^, Sophie Morales^1,2^, Xavier Gidrol^1,3,4^, Nathalie Picollet-D’hahan*^1,3,4^, and Cédric Allier*^1,2^

^1^Université Grenoble Alpes, Grenoble, F-38000, France

^2^Commissariat à l’énergie atomique et aux énergies alternatives, Laboratoire d’électronique et de technologie de l’information, Grenoble, F-38054, France

^3^Commissariat à l’énergie atomique et aux énergies alternatives, Biologie à Grande Echelle, Grenoble, F-38054

France

^4^Institut national de la santé et de la recherche médicale, U1038, Grenoble, F-38054, France

*Corresponding authors, contributed equally: cedric.allier@cea.fr, [nathalie.picollet-dhahan@cea.fr](mailto:nathalie.picollet-dhahan@cea.fr)

Movies description

Movie S1: Time-lapse acquisition of the first experiment of a 3D culture of RWPE-1 cells monitored with the lens-free tomographic setup. The views are orthogonal average intensity projection of the reconstructed volume. The regularized Gerchberg-Saxton algorithm was run with the following parameters:$\varphi\in\left\{ 0^{\circ},305^{\circ} \right\}$,$\Delta\varphi=9.8^{\circ}, \theta=45^{\circ}$, 3D volume = $2.67\times2.67\times0.68=4.86$ mm^3^,  $800\times800\times128$ voxels of $3.34\times3.34\times5.32=60 \mu m$^3^. The clusters of cells have been color-coded to visualize their position along the different axis of projection. The color gives the depth in the volume for each view. The blue color encodes for the highest positions ($z=0.34$ mm for the $xy$-view, $x=-1.34$ mm for the $yz$-view, $y=1.34$ mm for the $xz$-view) and the red color encodes for the deepest positions ($z=-0.34$ mm for the $xy$-view, $x=1.34$ mm for the $yz$-view, $y=-1.34$ mm for the $xz$-view). Time is given as HH:MM.

Movie S2: Time-lapse acquisition of three clusters of cells. The inverse problem algorithm was run with the following parameters: $\varphi\in\left\{ 0^{\circ},305^{\circ} \right\}$,$\Delta\varphi=9.8^{\circ}, \theta=45^{\circ}$, 3D volume = $0.42\times0.42\times0.21=0.036$ mm^3^,  $256\times256\times79$ voxels of $1.67\times1.67\times2.66=7.4 \mu m$^3^. On each view, the colors codes for the depth: the shallowest in blue, the deepest in red.Time is given as HH:MM.

Movie S3: Time-lapse acquisition of a region of interest showing the growth of a cluster of cells initiated with the merging of three single cells that were first orbiting around each other. This cluster is shown outlined in green. At $t_{0}$+95 h, this newly formed cluster generated three extensions of 70 $\mu m$, 220 $\mu m$ and 190 $\mu m$ in length. These branching cells were finally re-absorbed and, doing so, the cluster attracted to itself a large number of cells present in its neighboring. In total this cluster has grown through the merging of 15 clusters that were initially present within a circle of 450 $\mu m$ in diameter. For instance at $t_{0}+120 h$, this aggregate attracted to itself two other cell clusters that were at distances of about 100 $\mu m$. This cell aggregate became unstable. By three times it ejected small clusters of cells, strikingly in the direction of another cell aggregate located at a distance of 250 µm. The reconstruction parameters are identical to that of movie S1. Time is given as HH:MM.

Movie S4: Time-lapse acquisition of a region of interest showing the merging of two cell aggregates consecutive to apparent pairwise attraction. These two clusters are shown outlined in green.

At $t_{0}+68 h$, the two aggregates are about 55 $\mu m$ in diameter and they are separated by a distance of 450 $\mu m$. They first moved towards each other, accumulating cells from the surroundings. When the distance between the two aggregates was about 100 $\mu m$, branching cells emerged that connected the two aggregates before the final merging. The resulting aggregate continued to move a little, by 150 $\mu m$ and accumulated some neighboring cells. Finally at $t_{0}+280 h$ this aggregate was as large as 140 $\mu m$ in diameter. The reconstruction parameters are identical to that of movie S1. Time is given as HH:MM.

Movie S5: Time-lapse acquisition of a region of interest showing the accumulation of 25 clusters of cells into one very large aggregate of ~6000 $\mu m^{2}$ ($xy$-projected area) that next managed to move as a cohesive group at a speed of 5 to 10 µm/h. The large aggregate is shown outlined in green. At $t_{0}+184 h$ hours, following a large expansion of the cell aggregate, we observed a traction force generated by this aggregate onto the ECM. This can be indirectly observed through the displacement of several clusters of cells towards the aggregate. These tractions forces disappeared as the aggregate released the tensions applied to the ECM and the clusters moved then back to their initial positions. The inverse problem algorithm was run with the following parameters: $\varphi\in\left\{ 0^{\circ},305^{\circ} \right\}$,$\Delta\varphi=9.8^{\circ}, \theta=45^{\circ}$, 3D volume = $1.34\times1.34\times0.53=1$ mm^3^,  $800\times800\times200$ voxels of $1.67\times1.67\times2.66=7.4 \mu m$^3^. On each view, the colors codes for the depth: the shallowest in blue, the deepest in red. Time is given as HH:MM.

Movie S6: Time-lapse acquisition of the second experiment of a 3D culture of RWPE-1 cells monitored with the lens-free tomographic setup. This 3D culture of RWPE-1 cells featured 10 μm beads embedded into the ECM. The views are orthogonal average intensity projection of the reconstructed volume. The regularized Gerchberg-Saxton algorithm was run with the following parameters:$\varphi\in\left\{ 0^{\circ},305^{\circ} \right\}$,$\Delta\varphi=9.8^{\circ}, \theta=45^{\circ}$, 3D volume = $2.67\times2.67\times0.78=5.57 \mathrm{mm}^{3}$, $800\times800\times128$ voxels of $3.34\times3.34\times6.10=68 \mu m^{3}$. The clusters of cells have been color-coded to visualize their position along the different axis of projection. The color gives the depth in the volume for each views. The blue color encodes for the highest positions ($z = 0.37$ mm for the $xy$-view, $x = -1.34$ mm for the $yz$-view, $y = 1.34$mm for the $xz$-view) and the red color encodes for the deepest positions ($z = -0.37$mm for the $xy$-view, $x = 1.34$mm for the $yz$-view, $y = -1.34$mm for the $xz$-view). The $xz$-projections show clearly the presence of the 10 $\mu m$ beads embedded into the ECM. Time is given as HH:MM.

Movie S7: Time-lapse acquisition of a region of interest showing the dissociation at $t_{0}+53 h$ of a cell cluster into a fixed cluster and a migrating cluster. The latter moved along a linear path directly toward another cluster set a distance of 450 $\mu m$. This initial path is next taken by several cells, migrating between the two fixed clusters, in both directions. And at $t_{0}+120 h$, the two fixed clusters are connected by a branch of cell of 200 $\mu m$ in length. The reconstruction parameters are identical to that of movie S6. Time is given as HH:MM.

Movie S8: Time-lapse acquisition of a region of interest showing the total dissociation of a cluster of four cells that further migrated as single cells at a speed of about 30 $\mu m/h$. These cells migrated along linear paths, they followed each other, sometimes turning back. Importantly they formed trains of cells moving as cohesive groups and in a second phase several paths taken by the trains of cells became gradually branches of cells connecting two fixed clusters separated by ~300 $\mu m$. The reconstruction parameters are identical to that of movie S6. Time is given as HH:MM.

Movie S9: Time-lapse acquisition of a region of interest showing ECM deformation resulting from traction forces generated by a cell aggregate. The views are the 3D orthogonal projections. The reconstruction parameters are identical to that of movie S6. Time is given as HH:MM.

Cooling system

In order to control the temperature of the cell culture, an air flow is brought through the sample holder to create a thermal insulation of the cell culture with an air knife between the sensor and the Petri dish. The air flow is obtained through pressurized air. No ventilator nor turbine are used in order to limit the amount of vibrations caused by this cooling system.

Without the cooling system, the temperature inside the Petri dish reaches about 48 °C in less than 45 min and condensation occurs on the cap preventing any acquisition (see Fig. S1). When the cooling system is activated, the temperature quickly drops to its initial value in less than 15 min. When acquisitions are performed, as the sensor rotates, the geometry of the air knife changes, lowering the efficiency of the cooling system. This leads to small bumps in the temperature regulation. They nevertheless remain confined within a 0.2 °C range, which is adapted for cell culture.


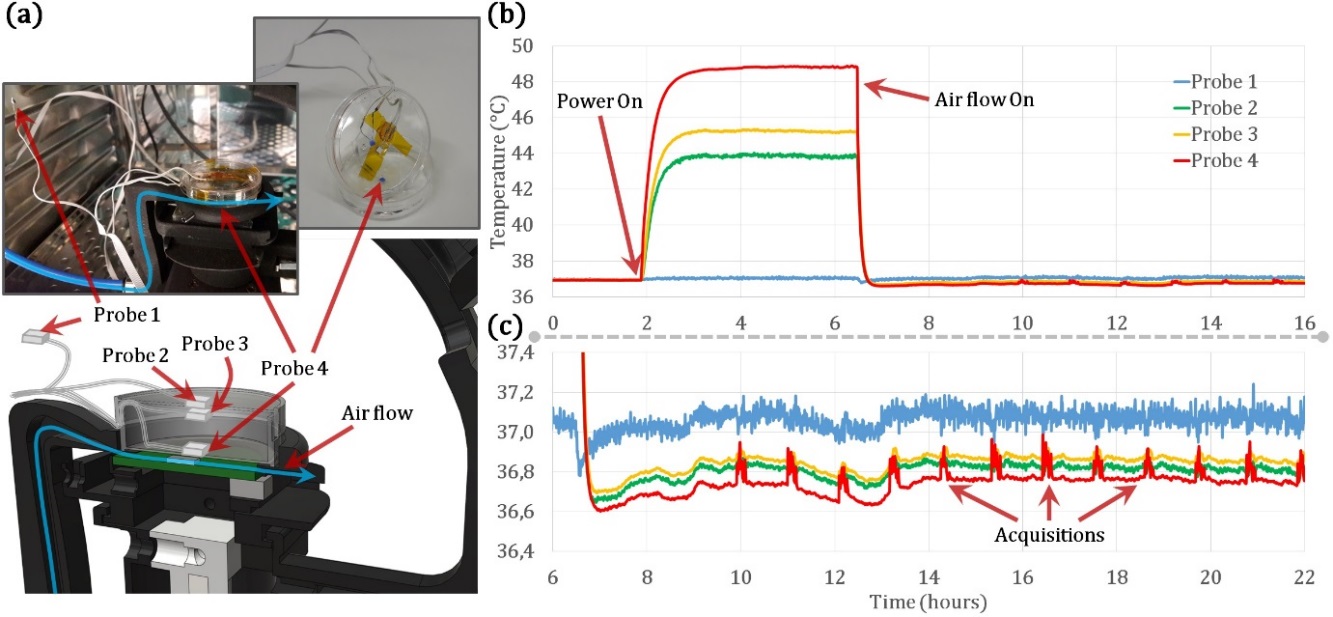


Figure S1. (a) Scheme of the cooling system: an air flow is brought through the sample holder to create a thermal insulation of the cell culture with an air knife between the sensor and the Petri dish. To test the efficiency of the system, thermal probes were placed on a mock Petri dish (35 mm diameter) at different places: probe 1 in the incubator, probe 2 at the top of the cap, probe 3 in the dish at the bottom of the cap and probe 4 at the bottom of the dish. (b) Temperature curves of the four probes during the mock experiment: the system is placed into the incubator and at t =2 h the power is turned on. As the sensor warms up, the temperature in the Petri dish increases rapidly. At t=6.5 h, the cooling air flow is triggered and the temperatures drop to standard culture values. (c) Focus on temperature curves during the acquisition process: starting at t=10 h an acquisition lasting 15 minutes is performed every hour.

Holographic reconstruction

In the lens-free microscope geometry, the phase information of the total wave $U_{tot}$ has to be recovered from the measured intensity ${I_{d}=\left| U_{tot} \right|}^{2}$. $U_{tot}$ is the interference of the incident wave $U_{inc}$ produced by the LED and the diffracted wave $U_{dif}$ scattered by the sample (see Fig. 1a and Fig. S2a): $U_{tot}=U_{inc}+U_{dif}$. A given dataset is consequently composed of a set of $N$ intensities $I_{d}^{j}, j\in\left\| 1,N \right\|$ recorded at different illumination angles $\left( \theta_{j},\varphi_{j} \right)$ and at different wavelengths$\lambda_{j}$. The 3D diffracting sample is described by its scattering potential *f* defined at each point of space $\vec{r}=\left( x,y,z \right)$by:

$f\left( \vec{r} \right)=\left( \frac{n\left( \vec{r} \right)}{n_{0}} \right)^{2}-1$ (1)

where $n(r)$ is the local complex refractive index and $n_{0}$ is the refractive index of the surrounding medium. In the hypothesis of transparent objects, we assume that the scattering potential is the same regardless of the illumination wavelength. For a monochromatic incident plane wave $U_{inc}=e^{ik_{0}.\vec{r}}$ of wave vector $\vec{k_{0}}=\frac{2\pi n_{0}}{\lambda}(p_{0},q_{0},m_{0})$ and under the first-order Born approximation, the Fourier diffraction theorem ^12^ links the 3D Fourier transform of the scattering potential *f* with the 2D Fourier transform of the diffracted field $U_{dif}$ on a given plane at $z=z^{+}$(see Fig. S2):

$\hat{f}\left( \alpha,\beta,\gamma\right)=\frac{4\pi}{ik_{0}^{2}}w\left( u,v \right)e^{-2i\pi w\left( u,v \right)z^{+}}\hat{U}_{dif}\left( u,v;z^{+} \right)$ (2)

where $\left( u,v,w \right)$ and $\left( \alpha,\beta,\gamma\right)$ are respectively the coordinates in the 2D Fourier space on the plane $z = z^{+}$ and in the 3D Fourier space of the object. They satisfy the following relationships:

$\left\{ \begin{matrix} \alpha=u-u_{0} \\ \beta=v-v_{0} \\ \gamma=w-w_{0} \end{matrix} \right.$ and $w\left( u,v \right)=\sqrt{\frac{n_{0}^{2}}{\lambda^{2}}-u^{2}-v^{2}}$ with $\left( u_{0},v_{0},w_{0} \right)=\frac{n_{0}}{\lambda}\left( p_{0},q_{0},m_{0} \right)$ (3)

The Fourier transform and its inverse transform are here defined for a given function $g$ as:

$\mathcal{F}\left( g \right)\left( u \right)=\hat{g}\left( u \right)=\int_{-\infty}^{\infty} g\left( x \right)e^{-2i\pi ux}dx$ and $\mathcal{F}^{-1}\left( \hat{g} \right)\left( x \right)=\int_{-\infty}^{\infty} \hat{g}\left( u \right)e^{2i\pi xu}du$ (4)

As shown schematically on Fig. S2, Eq. (3) implies that the two-dimensional surface $\hat{U}_{dif}$ is mapped on a spherical cap (the so-called Ewald’s sphere) the 3D Fourier space. This cap depends on $\vec{k_{0}}$ which gives its orientation via the direction $(p_{0},q_{0},m_{0})$ and its radius via the wavelength $\lambda$. The Born approximation used in (2) is only valid for weakly scattering objects. This condition is not met with 3D cell cultures where objects are larger than a few tens of microns. For a single cell with a typical refractive index of 1.36 in water ^3^, the relative refractive index is $\delta n\sim0.03$. With a typical cell length of $l\sim20 \mu m$ and in the visible light $\lambda\sim550$ nm the overall introduced phase delay is $\frac{2\pi}{\lambda}.l.\delta n>2\pi$ which is not negligible. Nonetheless, the Born approximation and the Fourier diffraction theorem will be used in our 3D reconstruction algorithms to determine some morphological information on the biological sample, such as their positions, dimensions and shapes. But it cannot be expected that 3D reconstructions are phase quantitative. The Fourier diffraction theorem can be used both for simulation of the diffracted wave $U_{dif}$ from a numerical $f$ (Fig. S2a - clockwise) and for direct reconstruction of $f$via mapping of the Fourier coefficients of $\hat{f}$ (Fig. S2b – counter-clockwise)*.* Nevertheless, the reconstruction is ill-posed as it requires access to both the amplitude and the phase of the different diffracted waves $U_{dif}^{j}$ whereas only the intensity of the total wave $I_{d}=\left| U_{tot} \right|^{2}$ is recorded (Fig. 1a). Here we have used two different phase retrieval techniques to reconstruct the 3D experimental datasets (Figs. 1cd). The first 3D reconstruction technique is based on a regularized Gerchberg-Saxton algorithm (Fig. S3a and S4a) in which back and forth propagations between the experimental data and the 3D reconstructed object are performed to insure data fidelity with constraints and regularizations applied to the 3D diffracting objects. The second 3D reconstruction technique is based on an inverse problem approach (Fig. S3b and S4b) using the Fourier diffraction theorem ^2^ as a direct model. The two algorithms are able to overcome the limitations raised by the lens-free microscope, *i.e.* the lack of phase information and the limited angular coverage. They recover the overall shape and the positions of the 3D cellular objects. The algorithm based on the 3D inverse problem approach gives the best results in terms of contrast and quality. There are however time consuming and are used only to reconstruct small region of interests at full resolution. For the reconstruction of volume as large as ~5 mm^3^, we used the Gerchberg-Saxton algorithm which is faster and provide sufficient quality results.


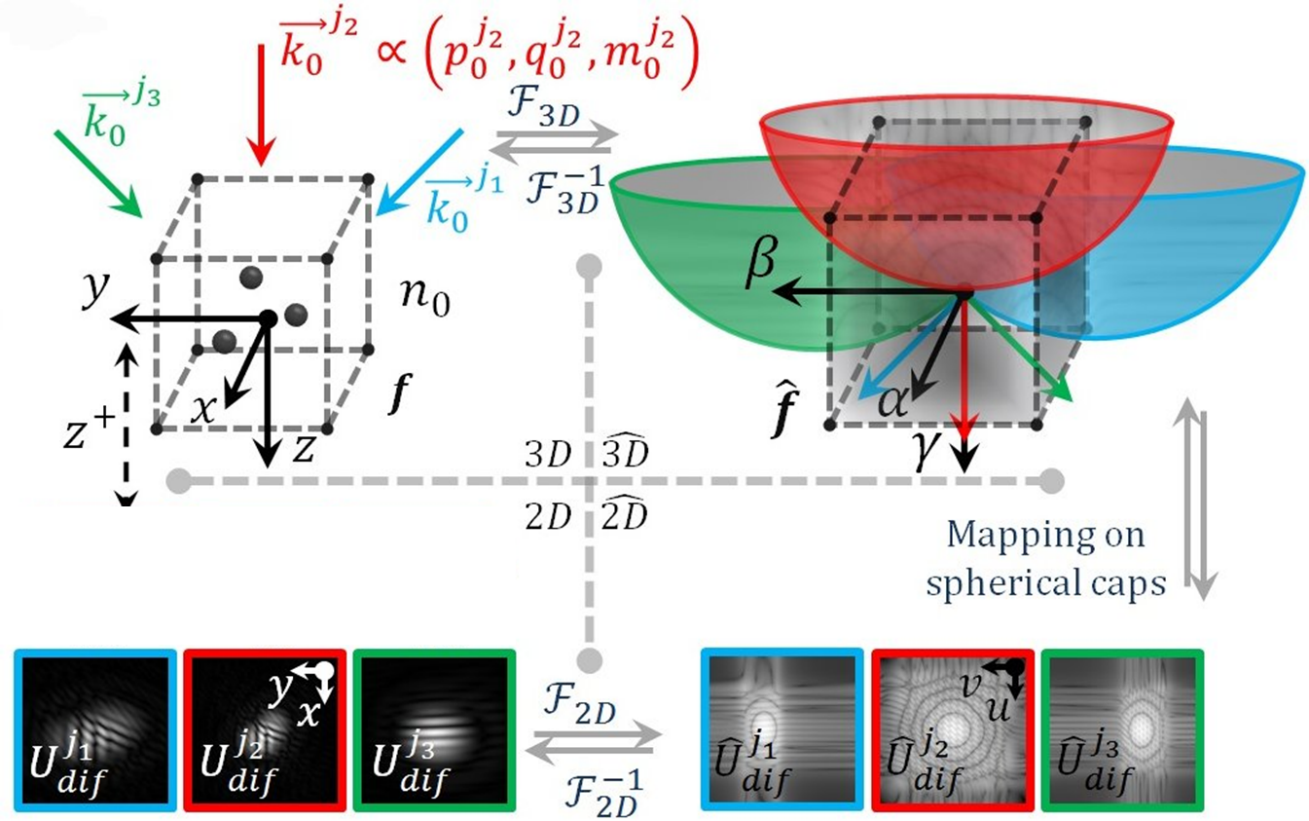


Figure S2. Geometrical illustration of the Fourier diffraction theorem: for each lighting situation j the 2D Fourier transform of the diffracted wave $U_{dif}^{j}$ is mapped on spherical caps in the 3D frequency domain of the scattering potential $f$. The orientation and radius of these caps directly depend on the illumination directions ${\vec{k_{0}}}^{j}\propto(p_{0}^{j},q_{0}^{j},m_{0}^{j})$ and the associated wavelength $\lambda^{j}$.


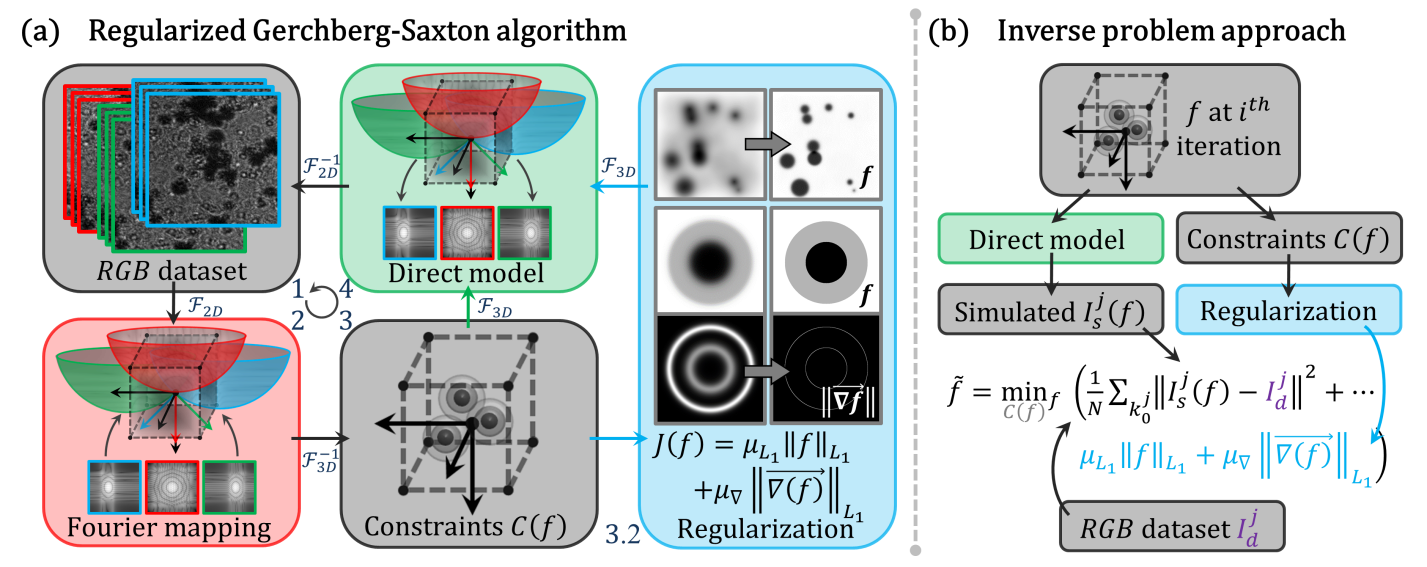


Figure S3. Scheme of the two 3D reconstruction algorithms.

1. Scheme of the regularized Gerchberg-Saxton algorithm. Step 1: The algorithm is initialized with a set of complex waves. Their modulus is directly the square root of the recorded intensities. Their unknown phase is initialized to 0. Step 2: The 3D Fourier domain of the object $\hat{f}$ is mapped using the Fourier diffraction theorem. Step 3: Domain constraints are applied on the simulated object. Step 4: The new object is used to simulate a new set of complex waves using the Fourier diffraction theorem as a direct model. Next, the algorithm loops to start a new iteration. Data fidelity is performed by keeping the simulated phase and replacing the simulated modulus by the recorded dataset. In between the steps 3 and 4, an additional step can be added: Step 3.2. After applying the constraints, the object is regularized with a sparsity constraint on the object and its gradient.
2. Scheme of the inverse problem approach. This approach consists in minimizing a cost function. At each iteration of the algorithm, this cost function is computed using the simulated object. The direct model is used to simulate the corresponding intensities $I_{s}^{j}$. They are compared with the recorded intensities $I_{d}^{j}$ to create a data fidelity term. The two other terms of the cost function, in blue, characterizes the sparsity of $f$ and of its gradient.


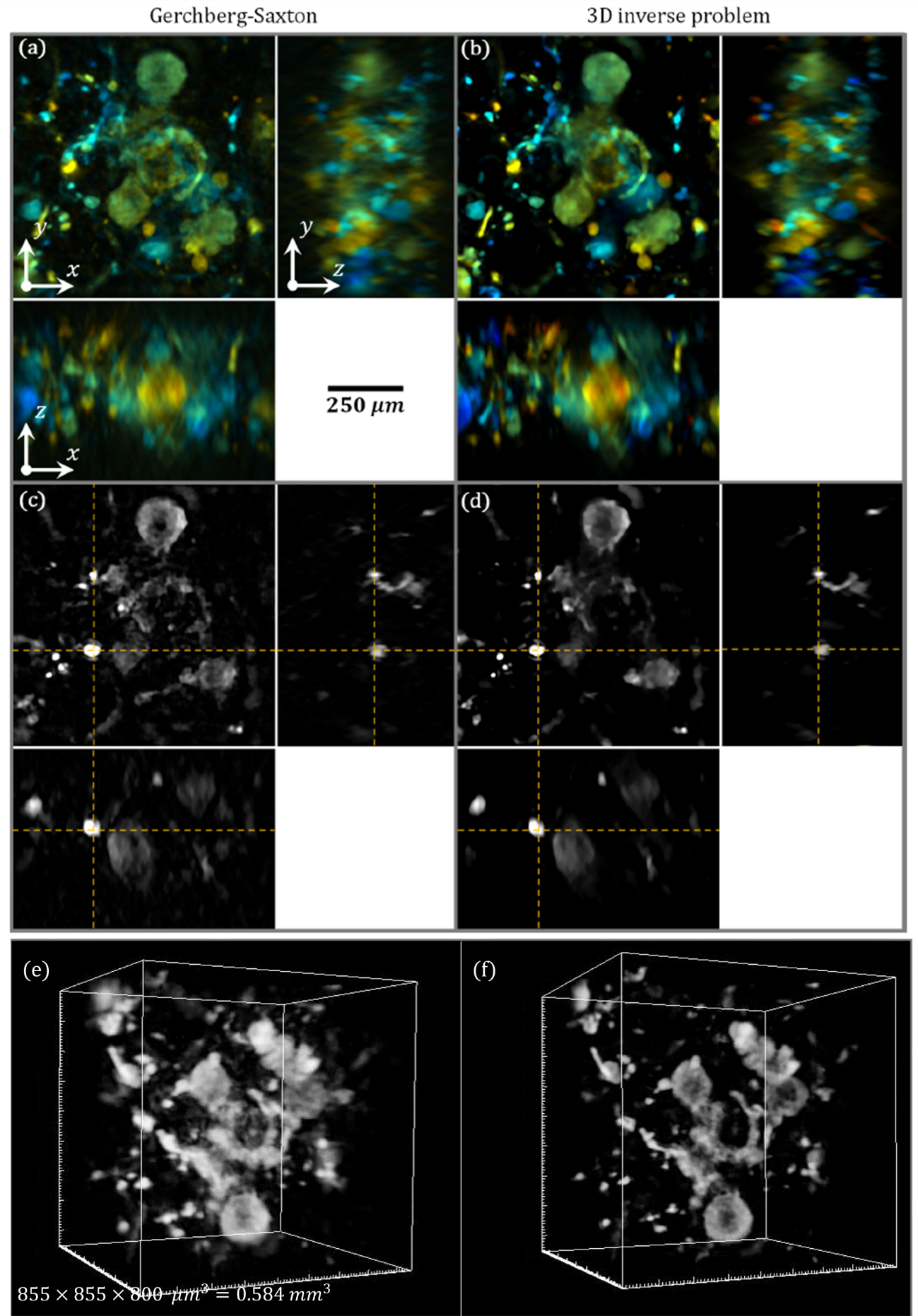


Figure S4 Visualizations of the reconstructed volume of a 3D cell culture RWPE-1 cells. The regularized Gerchberg-Saxton algorithm and the inverse problem approach were run with the following paramaters: $3\times31$ *RGB* acquisitions with 31 angles$\varphi\in\left\{ 0^{\circ},282^{\circ} \right\}$,$\Delta\varphi=9.4^{\circ}$ and $\theta=45^{\circ}$, 3D volume$=855\times855\times800=0.584$ mm^3^, $512\times512\times300$ voxels of $1.67\times1.67\times2.66 \mu m^{3}$. (a-b) Orthogonal average intensity projections. On each view, the colors codes for the depth: the shallowest in blue, the deepest in red. (c-d) Orthogonal slices: $xy$-slice at $z = 48 \mu m$, $xz$-slice at $y = -117 \mu m$, $yz$-slice at $x = -164 \mu m$. (ef) 3D view of the reconstructed volume.

*Regularized Gerchberg-Saxton algorithm*

Figure S3 shows the scheme of the Gerchberg-Saxton algorithm ^4^ which was developed. It presents two sub-algorithms. The first algorithm is a standard Gerchberg-Saxton algorithm in which a constraint is applied on the 3D objects ^5^. It computes an iterative loop featuring 4 steps. Step 1: At the first iteration it = 0 of the algorithm, the complex waves on the sensor at the different illumination $j\in\left\| 1,N \right\|$ are initialized with moduli corresponding to the recorded intensities and the phase set to 0: $U_{tot,0}^{j}=\sqrt{I_{d}^{j}}.$ Further iterations insure data fidelity by replacing in the modulus of complex waves simulated in step 4 with the square root of the recorded intensity:

$U_{tot,it}^{j}=\frac{\sqrt{I_{d}^{j}}}{\left| U_{tot,it-1}^{j} \right|}U_{tot,it-1}^{j}$ (5)

Step 2: Back-propagation of the complex wave from the sensor plane to the reconstructed volume. To this aim the 3D Fourier domain $\hat{f}$is mapped with the corresponding diffracted waves $U_{dif, it}^{j}$ according to the Fourier diffraction theorem ^12^. The object $f$ is obtained through the inverse 3D Fourier transform of $\hat{f}$. Step 3: Constraints of positivity are applied on the real and the imaginary parts of the updated retrieved object $f$. Indeed, for weakly scattering object, $\left| \delta n \right|=\left| n-n_{0} \right|\ll1$, Eq. (1) gives: $f\sim2\delta n/n_{0}$. And the objects are transparent ($\mathfrak{R}\left( \delta n \right)>0$) or absorbing ($\mathfrak{I}\left( \partial\delta n \right)>0$). Step 4: Using the Fourier diffraction theorem as direct model, a new set of complex waves $U_{tot, it}^{j}$ is simulated.

Given the microscope geometry, the angular coverage of the sample is very limited. This algorithm is not efficient to properly compensate this lack of information in the dataset. It is consequently only used on a few iterations to initialize the reconstruction. Then, a new step is added in the loop, between steps 3 and 4, leading to the blue loop in Fig S3.a. This step 3.2 consists of regularizing the 3D object $f$. To do so, a minimization problem is defined as:

$\tilde{\delta}t=\underset{C\left( f \right)}{\mathrm{argmin}} \mu_{L_{1}}\left\| f \right\|_{L_{1},\epsilon}+\mu_{\nabla}\left\| \nabla f \right\|_{L_{1},\epsilon}$ (6)

The L1-norm being here defined for a given 3D complex matrix $M$ as:

$$\left\| M \right\|_{L_{1},\epsilon}=\frac{1}{nb_{x}nb_{y}nb_{z}}\sum_{k,l} \sqrt{\left| M_{k,l,m} \right|^{2}+\epsilon^{2}}$$

$$\left| \nabla M_{k,l,m} \right|^{2}=\frac{1}{4}\left[ q_{x}\left| M_{k+1,l,m}-M_{k,l,m} \right|^{2}+q_{x}\left| M_{k+1,l+1,m}-M_{k,l+1,m} \right|^{2}+q_{x}\left| M_{k+1,l,m+1}-M_{k,l,m+1} \right|^{2}+ \right.q_{x}\left| M_{k+1,l+1,m+1}-M_{k,l+1,m+1} \right|^{2}+q_{y}\left| M_{k,l+1,m}-M_{k,l,m} \right|^{2}+q_{y}\left| M_{k+1,l+1,m}-M_{k+1,l,m} \right|^{2}+q_{y}\left| M_{k,l+1,m+1}-M_{k,l+1,m+1} \right|^{2}+q_{y}\left| M_{k+1,l+1,m+1}-M_{k+1,l,m+1} \right|^{2}+q_{z}\left| M_{k,l,m+1}-M_{k,l,m} \right|^{2}+\left. q_{z}\left| M_{k+1,l,m+1}-M_{k+1,l,m} \right|^{2}+q_{z}\left| M_{k,l+1,m+1}-M_{k,l+1,m} \right|^{2}+q_{z}\left| M_{k+1,l+1,m+1}-M_{k+1,l+1,m} \right|^{2} \right]$$

(7)

where the indices $\left( k,l,m \right)$ stand for the $\left( {nb}_{x}, {nb}_{y},{nb}_{z} \right)$voxels locations on the volume grid respectively on the $x$ and $y$ and $z$ axes. $\epsilon$ is a small number to ensure the differentiability of the L1-norm in the vicinity of $\left( q_{x}, q_{y},q_{z} \right)$ are weighting coefficients to take into account that the grid can be not orthonormal. In our case, tests showed that these parameters do not influence much the reconstructions and for simplicity, they were set to: $q_{x}= q_{y}=q_{z}=1$. In Eq. (6), $C(f)$ stands for the constraint given on the domain of admissible solutions. The first term is a sparsity prior. For sparse objects, it aims at enforcing a reconstructed volume mainly composed of isolated ”particles” ^67^. The second term is an edge-preserving regularization ^89^ via a sparsity prior on the gradient of $f$. It enforces smooth and almost uniform volumes while preserving sharp edges. Two hyperparameters $\mu_{L_{1}}$and $\mu_{\nabla}$ are used to appropriately tune the trade-off between these two regularization terms in (6). In all the presented reconstructions, a fixed ratio of $\mu_{L_{1}}/\mu_{\nabla}=1.25$was used. The regularization consists in minimizing the criterion (6), which is performed by a variable metric method with limited memory requirements and bound constraints (VMLMB) algorithm ^10^, a modified limited memory quasi-Newton convex optimization method with Broyden–Fletcher–Goldfarb–Shanno (BFGS) updates and bound constraints. The aim of this regularization is to reduce the artefacts of the reconstructions due to the lack of phase information in the dataset recorded by the sensor and to the limited angular coverage. To accelerate the cleaning of the artefacts, one can note that there is no data fidelity term in the cost function (6). The data fidelity is only ensured later in the loop by step 4. As a consequence, a trivail solution of the minimization problem (6) exists, $f=0$. Thus, a limited number of iterations $nb_{reg}=10$is performed, on which the sparsity prior efficiently cleans the noise and artefacts with low values while the edge-preserving regularization maintains high the signal on the objects limiting their convergence toward 0. With this algorithm, when the step 4 is reached, the data fidelity of the simulated complex waves can be strongly degraded. Indeed, the regularization step forces the objects to be localized and cleans the noise. This new set of simulated waves can consequently be used to refine the alignment of the experimental data. This improves the overall reconstruction of the object *f* . To do so, between step 4 and step 1, the experimental data are aligned with the simulated intensities via a least square minimization of the difference between the latter and the former for a given shift ^11^. This alignment step is time consuming and is not performed at each iteration of the loop.

*Inverse problem approach*

In the previous algorithm, even if the phase is retrieved, only the coefficients lying on the spherical caps covered by the dataset are mapped in the Fourier domain. A huge part of the Fourier domain is thus left at its initialization value: 0. Moreover, the reconstructed volumes are beyond the validity domain of the Born approximation used in (2). This means that the model cannot perfectly fit the data even using a phase retrieval algorithm. Consequently, to improve the reconstructions quality the constraints on the data fidelity must be relaxed. To do so, an inverse problem approach has been implemented (Fig. S3b), using the Fourier diffraction theorem as a direct model to simulate the holograms of a given 3D object $f$. This direct model is thus at the base of the inverse problem approach implemented to iteratively retrieve the 3D object $f$. Working directly on the full volume f theoretically allows retrieving more Fourier coefficients than the previous methods via an extrapolation of the missing frequencies which lie outside spherical caps thanks to a priori information. Moreover, such an approach appropriately deals with the lack of phase information to reduce artifacts as no assumption on the phase on the sensor plane is needed. Finally, the simulated data are compared with the experimental measurements but are not forced to perfectly match them. This can partially take into account that the model of the Fourier diffraction theorem, only valid for low scattering objects, cannot perfectly simulate the experimental data for big objects. As presented on Fig. S3b, the inverse problem approach consists in minimizing the new cost function:

$$\tilde{f}=\underset{C\left( f \right)}{\mathrm{argmin}} \left[ \frac{1}{N}\sum_{j=1}^{N} \left\| I_{d}^{j}-I_{s}^{j}\left( f \right) \right\|^{2}+\mu_{L_{1}}\left\| f \right\|_{L_{1},\epsilon}+\mu_{\nabla}\left\| \nabla f \right\|_{L_{1},\epsilon} \right]\mathrm{with}I_{s}^{j}\left( f \right)=\left| U_{inc}^{j}+U_{dif}^{j}\left( f \right) \right|^{2}$$

(8)

where $C(f)$ stands for the domain constraints of the solution $\tilde{f}$. This minimization problem is solved using a convex optimization algorithm ^10^. In addition to the two regularization terms, the first term of this new cost function corresponds to a relaxed data fidelity. This means that after a few iterations the data fidelity is not strictly enforced. As in the previous method, the simulated intensities $I_{s}^{j}(f)$ can be used to refine the experimental data alignment. This algorithm gives the best reconstruction quality ^12^ but is very slow as the computation of the gradient of the cost function at each iteration is time consuming. As a consequence, only a few numbers of iterations are performed. It is first initialized using the Gerchberg-Saxton algorithm.

Characterization of the 3D lens-free microscopy setup

The lens-free microscope is robust to incubator conditions and able to routinely monitor large 3D cell cultures. Moreover, the reconstruction quality is sufficient for analyzing the reconstructed volumes and retrieving quantitative information on the biological objects: position, shape, volume, speed, and so on. These features need first quantitative analysis of the reconstructed volumes and it was hence necessary to characterize the lens-free microscope and the associated reconstruction codes both in terms of accuracy and resolution on a known object. An experiment dedicated to the lens-free microscope calibration was run and specific codes were implemented to compare the 3D reconstruction of a reference object from lens-free acquisitions with acquisitions through a standard microscope. The reference object consisting of 10 μm fluorescent microbeads embedded into a small drop of Matrigel.

*Experiment and data processing*

At the center of Greiner petri dishes (Sigma-Aldrich), a small drop of Matrigel is deposited to serve as a bed of another small layer of Matrigel in which fluorescent microbeads with a diameter of 10 μm (Sigma-Aldrich) are mixed. It is then allowed to polymerize for 30 minutes at 37 °C before addition of cell culture medium. Matrigel deforms along time and to limit this effect, the dish is thus put in incubator for three days before the acquisitions. The final volume is consequently composed of fluorescent microbeads randomly spread in the three dimensions. The reference for the positions is obtained by fluorescence microscopy: z-stacks fluorescent images are then acquired using an AxioObserver.Z1 inverted microscope (Zeiss) with a N-Achroplan 5x/0.13 Ph0 air objective mounted with an AxioCam

503 monochrome digital camera. In this configuration, the effective pixel size given by the microscope is 0.9080 μm on 1936$\times$1460 pixels ($1.76\times1.33=2.33$ mm^2^), 856 slices are imaged with a scanning pitch of: $\delta z=1 \mu m$. Let’s note here that no immersion oil is used and that the translation stage moves in the air. The axial distances given by the microscope correspond to distances in the air.

All lens-free 3D datasets are composed of $3\times32$ acquisitions done at 32 different angles $\varphi\in\left\{ 0^{\circ}, 305^{\circ} \right\}, \Delta\varphi=9.8^{\circ}$ in the three available wavelengths of the RGB LED ($\lambda_{B}=450 \mathrm{nm}$; $\lambda_{G}=520 \mathrm{nm}$; $\lambda_{R}=640 \mathrm{nm}$). In each dataset, a region of interest of 1024\times 1024 pixels (~2.9 mm^2^) is selected and aligned. Both the fluorescence and lens-free acquisitions are performed outside the incubator. The air flow is activated to prevent the sensor from warming the dish and to avoid any condensation on the Petri dish cap during the lens-free acquisitions. As the objects to reconstruct are simple, the regularized Gerchberg-Saxton algorithm presented is sufficient. The reconstructed volumes are composed of $512\times512\times400$ voxels of 3.343 μm^3^ for a global volume of $1.7\times1.7\times1.3= 3.9$ mm^3^.

*Position accuracy*

Figure S5 shows a comparison between the fluorescence acquisition and the lens-free reconstruction without cap. On the fluorescence view, one can see the elongated signal around the beads due to the out-of-focus fluorescence. To characterize the lens-free reconstructions, the position of the beads must first be extracted from the fluorescence $z$-stack and the reconstructed volumes. The same method is applied to the two volumes. First, a top view of the $xy$-plane is computed by selecting the maximal value of the volumes along the $z$-axis to perform a maximum intensity projection (see figures S5.c-d). These views are used to determine the positions of the beads on the $xy$-plane. An algorithm refines these 2D xy-positions and determines the altitude z of each bead. Presented in figure S6, it consists in extracting a coring of the volume around each bead over the whole height of the volume. For each bead, one gets a volume similar to that in the white medallions in figures S6.a,c,e. The coring is extracted inside the blue perimeters. In each coring a specific pattern (presented in the white medallions in figures S6.b,d,f) is matched by a 3D cross-correlation performed in the Fourier space to extract the 3D position. The chosen matching pattern is an ellipsoid of characteristic dimensions of $r_{x} = r_{y} = r_{rad}$ and $r_{z}$. The base of the extracted coring is a square of side $r_{rad}^{ext}$. These dimensions depend on the resolution of the pixel and the dimensions of the beads pattern in each volume.

• Fluorescence z-stack:$r_{x} = r_{y} = r_{rad}=5 \mathrm{pix}, r_{z}=45 \mathrm{pix}, r_{rad}^{ext}=8 \mathrm{pix}$

• Lens-free reconstruction:$r_{x} = r_{y} = r_{rad}=2 \mathrm{pix}, r_{z}=7 \mathrm{pix}, r_{rad}^{ext}=4\mathrm{pix}$


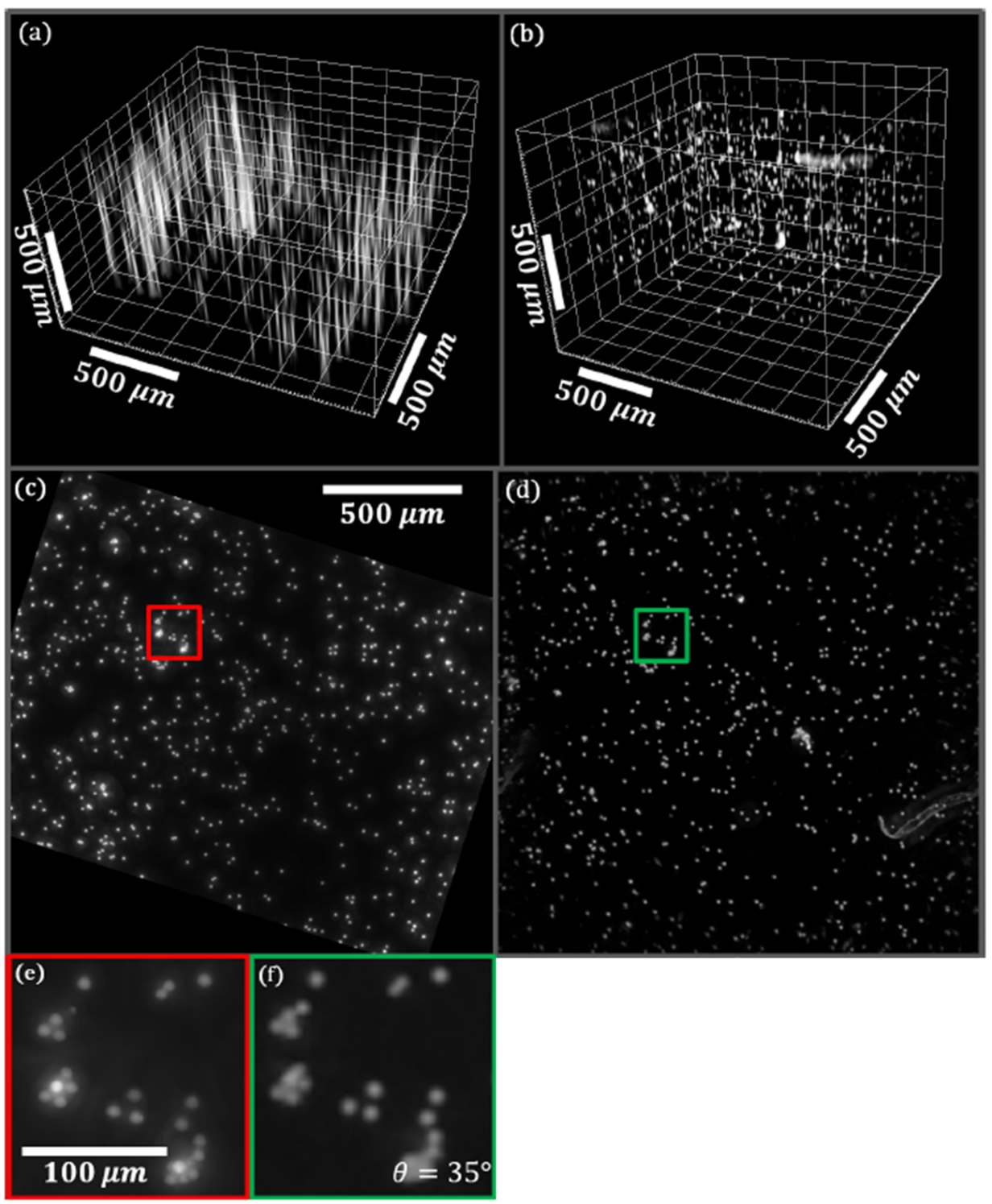


Figure S5. (a) Visualization of the z-stack acquired with the fluorescence microscope. (b) 3D lens-free view of the reconstructed scattering potential of the volume. (c) Maximum intensity projection of the volume presented in (a). (d) Maximum intensity projection of the volume presented in (b). (e) Zoom on a selected region of interest in the fluorescence view (c). (f) Zoom on the same region of interest in the lens-free view (d).


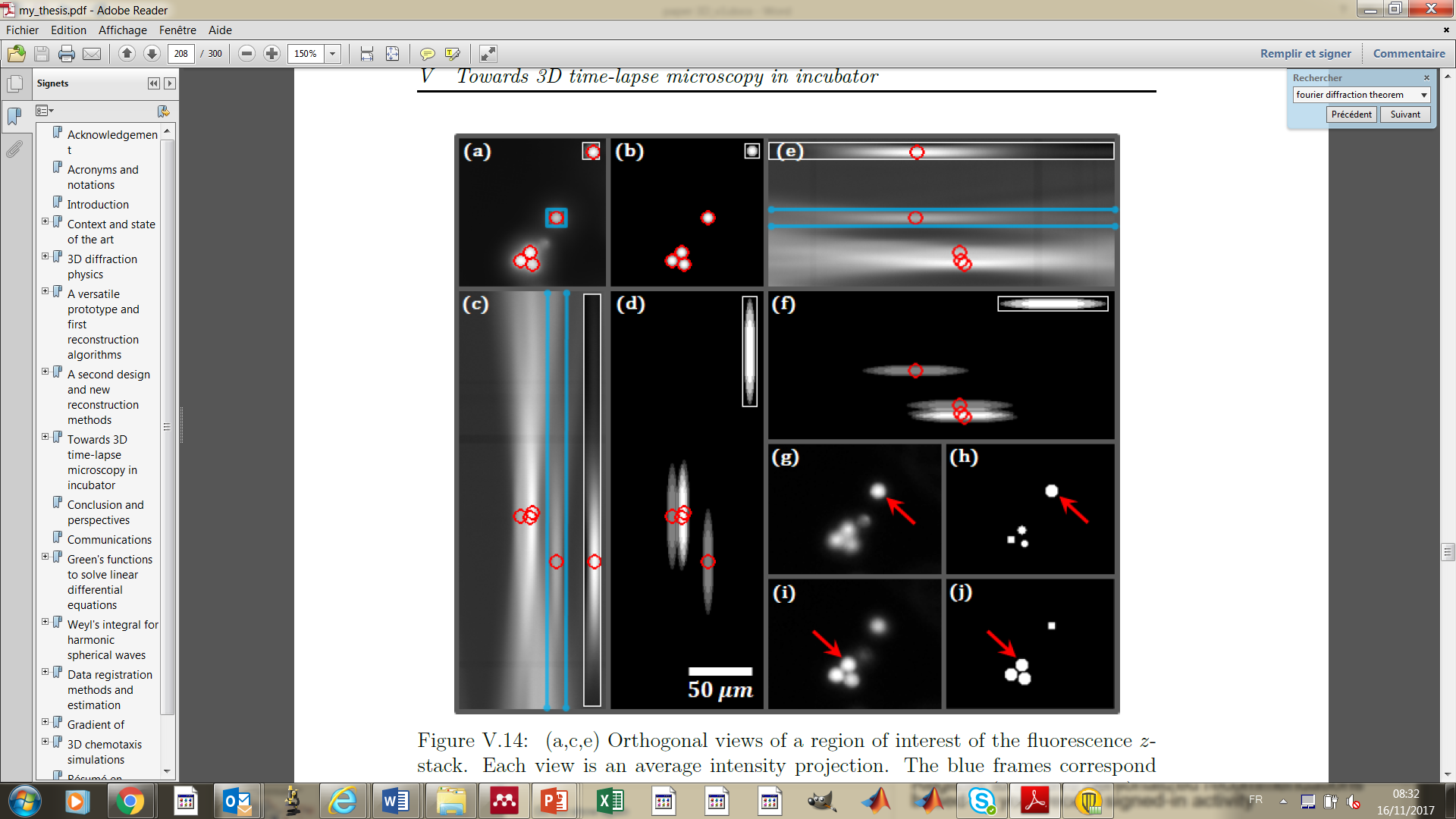


Figure S6. Orthogonal views of a region of interest of the fluorescence z-stack. Each view is an average intensity projection. The blue frames correspond to the side of the cored volume for a given bead. Its position (the red circles) is searched in this extracted volume (in the white medallions). (b,d,f) Orthogonal views of the region of interest synthesized with the found positions of the identified beads. The matching pattern is presented in the white medallions. (g-j) Comparison of the fluorescence z-stack (g,i) with the synthesized volume (h,j) at two different acquisition planes spaced$\Delta z=28 \mu m$. The red arrows point at beads which are in focus in a given plane.

For a checking purpose, the volumes are synthesized using the found 3D position and the chosen matching pattern. Comparisons between the original positions and the extracted position show a good agreement for both the fluorescence and the lens-free positions (figures S6.a,c,e,g,i versus b,d,f,h,j)). Once the 3D positions of the beads are known in the different volumes in terms of voxel, they must be paired. For each reconstructed lens-free volume, the positions of the beads ${\vec{r_{1}}}^{b_{i}}=(x_{1}^{b_{i}},y_{1}^{b_{i}},z_{1}^{b_{i}})$ extracted from the fluorescence z-stack are considered as the reference. These positions must be paired with the positions ${\vec{r_{2}}}^{b_{i}}=(x_{2}^{b_{i}},y_{2}^{b_{i}},z_{2}^{b_{i}})$ extracted from each of the lens-free reconstructions. To do so, the parameters of a transformation ${pos}_{1\to2}$between the coordinates of two given volumes must be determined. These parameters take in account different effects:

• $s_{2D}$: a scaling factor on the 2D xy-plane to deal with the difference in effective pixel size between the fluorescence acquisitions and the lens-free reconstructions.

• $s_{z}$: a scaling factor along the z axis to deal with the difference between the sampling z along the z-axis of the fluorescence acquisitions and the effective voxel size of the lens-free reconstructions.

• $\alpha$: the angle of rotation on the 2D xy-plane between the two volumes to deal with the 2D rotation of the sample between its position on the fluorescence and lens-free microscopes.

• $(x_{0},y_{0})$:the translation on the 2D xy-plane between the two volumes to deal with the 2D translation of the sample between its position on the fluorescence and lens-free microscopes.

• $z_{0}$: an offset on the z-axis to deal with a possible difference between the center of the volume acquired with the fluorescence microscope and the center of the reconstructed lens-free volumes.

In terms of 3D positions, this transformation works as follows:

$\left( \begin{matrix} x_{2}^{b_{i}} \\ y_{2}^{b_{i}} \\ z_{2}^{b_{i}} \end{matrix} \right)=pos_{1\to2}\left( \left[ s_{2D},s_{z},\alpha,x_{0},y_{0},z_{0} \right];\left( \begin{matrix} x_{1}^{b_{i}} \\ y_{1}^{b_{i}} \\ z_{1}^{b_{i}} \end{matrix} \right) \right)=\left( \begin{matrix} s_{2D} \\ s_{2D} \\ s_{z} \end{matrix} \right).\left( \begin{matrix} \cos\alpha& -sin \alpha& 0 \\ \sin\alpha& \cos\alpha& 0 \\ 0 & 0 & 1 \end{matrix} \right)\times\left( \begin{matrix} x_{1}^{b_{i}}-x_{0} \\ y_{1}^{b_{i}}-y_{0} \\ z_{1}^{b_{i}}-z_{0} \end{matrix} \right)$ (9)

Note here that the transformation (9) between the positions of the beads expressed in the reference frame of the first volume and their expression in the reference frame of the second volume does not take into account a possible tilt of the volume compared to the axial direction. This consequently assumes that for all the acquisitions, the sample remained strictly parallel to the sensor. Over the dimensions on the xy-axes larger than a few hundreds of microns, even a small tilt can lead to a difference of several tens of microns on the z-axis. This effect consequently degrades the expected lens-free accuracy found along the z-axis. To initialize the set of parameters, at least ${nb}_{b}$ = 6 matching pairs $\left\{ b_{1},b_{2},\ldots.,b_{n} \right\}$ are selected by the user. The set of parameters $\tilde{par}=\left[ \tilde{S_{2D},}\tilde{S_{2},}\tilde{\alpha},\tilde{x_{0}},\tilde{y_{0},}\tilde{z_{0}} \right]$ is then estimated by minimizing the distance between the transformed positions and the extracted positions in the second volume:

$\tilde{par}=\underset{par}{\mathrm{argmin}} \frac{1}{nb_{b}}\sum_{i=1}^{nb_{b}} \left\| {\vec{r_{2}}}^{b_{i}}-pos_{1\to2}\left( par;{\vec{r_{1}}}^{b_{i}} \right) \right\|_{2}$ (10)

Using this set of parameters, another program automatically proposes new matching pairs that the user can choose to keep or not. It is possible also to refine the set of parameters running the minimization problem again on the new set of matching pairs. As soon as the user estimates that enough pairs have been chosen and that the automatic matching is satisfactory, a last refinement of the parameters is done before an automatic matching of all the beads. For each bead identified in the first volume, the automatic matching consists in computing their corresponding positions in the second volume from their position in the first volume via equation (9) and finding the closest identified bead in the second volume. If the distance between this closest bead and the computed position falls within a tolerance of 20 voxels, the two beads are paired. Otherwise, the bead is put apart. If the bead in the second volume is already paired, the bead in the first volume giving the closest computed position in the second volume is kept. Figure S7 presents the result for the pairing algorithm on the fluorescence $z$-stack and the lens-free reconstruction. Figure S8 presents the results on the beads which are paired between the fluorescence z-stack and the 3 lens-free volumes with three different possible illumination tilting angles $\theta\in\left\{ 35^{\circ}, 45^{\circ}, 55^{\circ} \right\}$without cap on the Petri dish. On the $xy$-plane, the data are not scaled but only rotated and translated to give the best matching between the clouds of points. The distances to the rotation center are directly given by the effective pixel size of the different volumes. Looking at the $xy$-view in figure S8a, the lens-free reconstruction seems dilated. A zoom on the bottom-right corner in figure S8d shows that this effect happens for the three tested angles but with a different coefficient. The higher is the angle, the bigger is the coefficient. On the z-axis the positions of the lens-free reconstructions are translated but also scaled to match the fluorescence positions. It seems to be a very good agreement between the paired positions. The zooms on the $xz$-view in figures S8.ef show that the sample is tilted clockwise around the $y$-axis. As mentioned in the previous section, this transformation is not taken in account by equation (9) and will lead to a bias in the determination of the z-accuracy which will be underestimated. To better quantify these effects, the distribution of the positions of the beads in the reconstructed volumes as a function of the positions in the fluorescence z-stack is fitted on the xy-plane and along the axial direction.


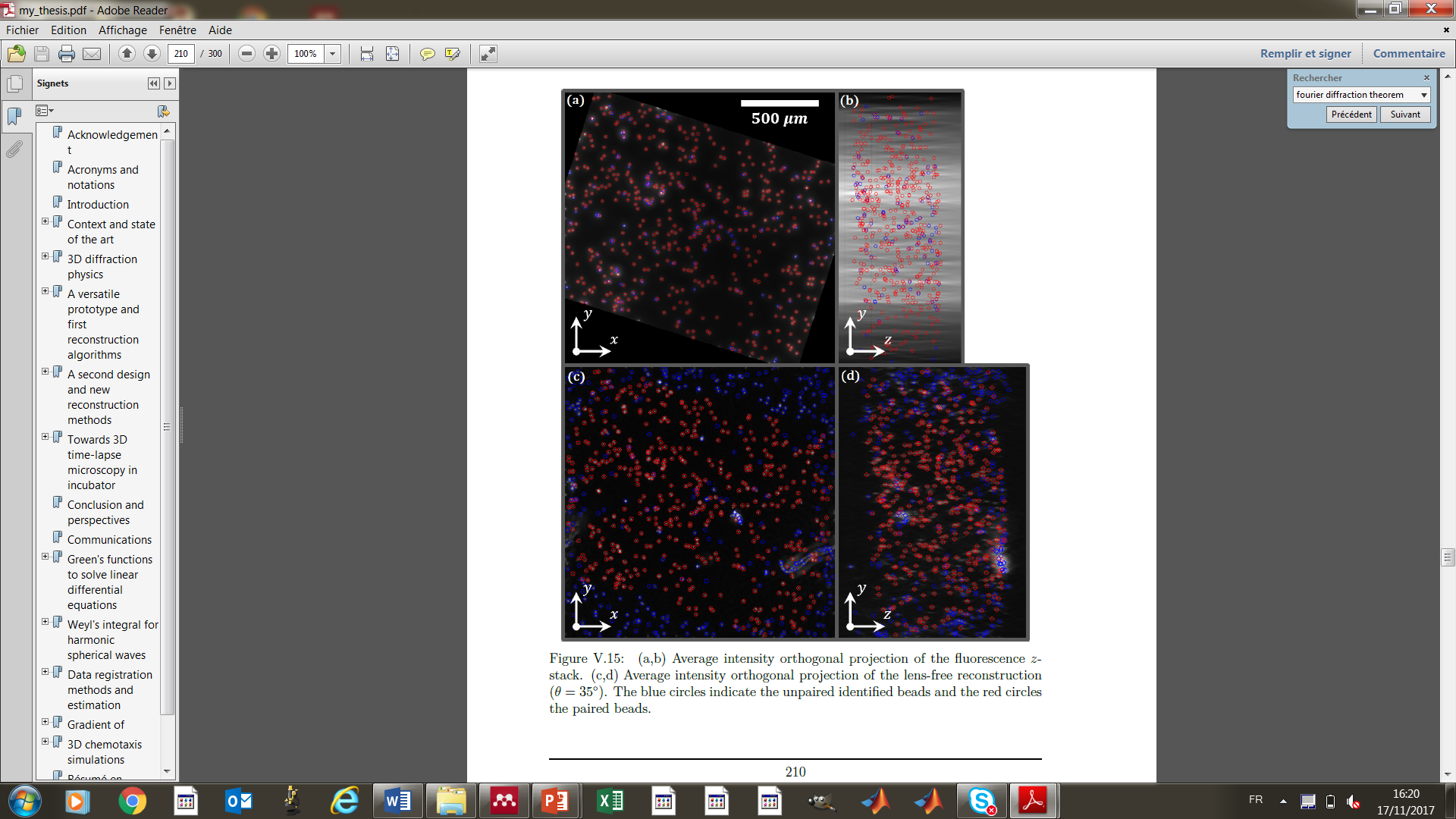


Figure S7. (a,b) Average intensity orthogonal projection of the fluorescence z-stack. (c,d) Average intensity orthogonal projection of the lens-free reconstruction. The blue circles indicate the unpaired identified beads and the red circles

the paired beads.


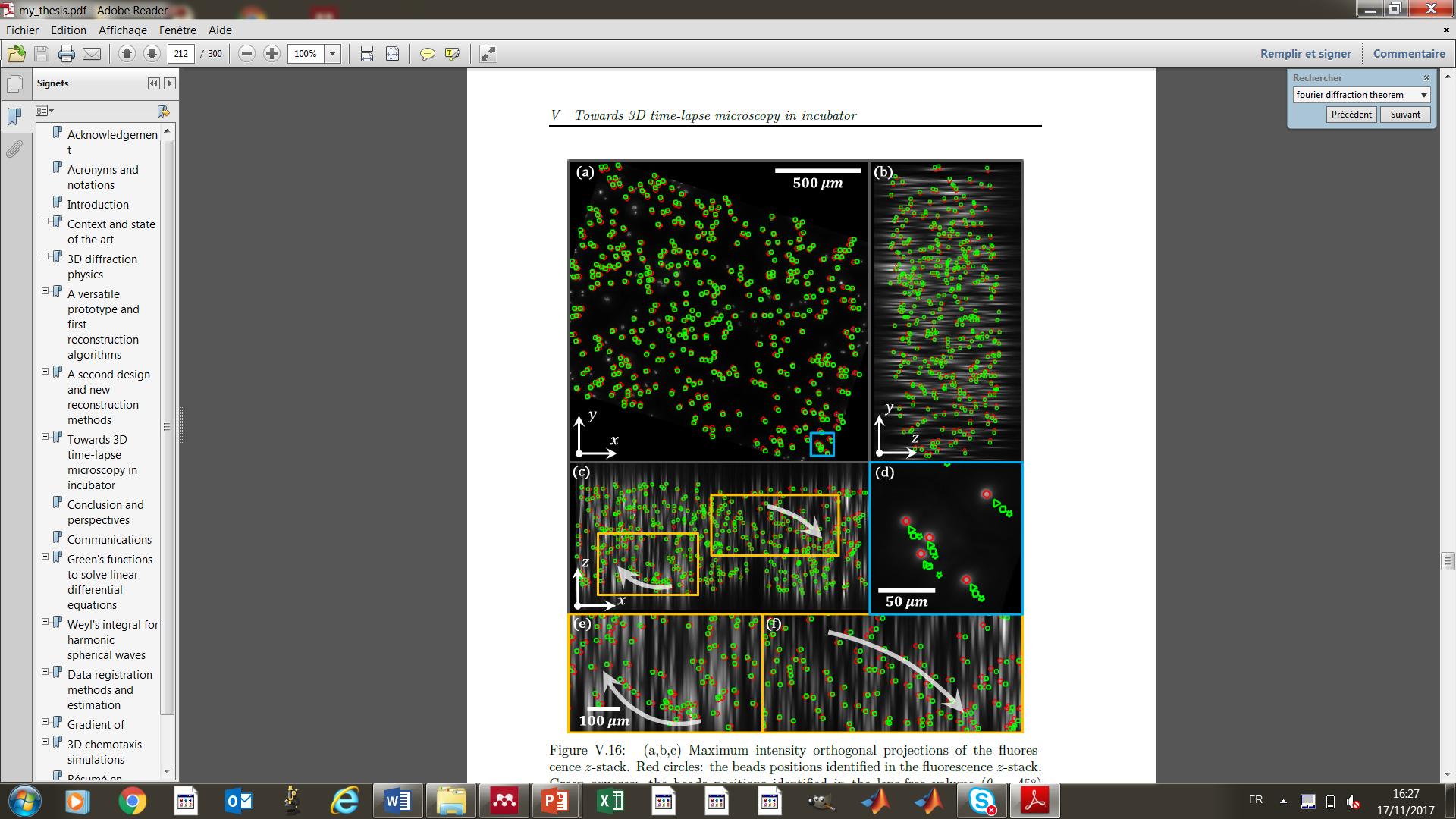


Figure S8. (a,b,c) Maximum intensity orthogonal projections of the fluorescence $z$-stack. Red circles: the beads positions identified in the fluorescence z-stack. Green squares: the beads positions identified in the lens-free volume ($\theta=45^{\circ}$). (d) Zoom on the blue square on (a). Red circles: the $xy$-positions in the fluorescence $z$-stack. Green markers: the $xy$-positions in the lens-free reconstructed volumes at $\theta=35^{\circ}$ (triangles), $\theta=45^{\circ}$ (squares) and $\theta=55^{\circ}$ (pentagons). (e-f) Zooms on the orange rectangles on the xz-view (c). The gray arrows emphasize a tilt angle of the Petri dish between the acquisitions on the fluorescence microscope and the lens-free microscope.


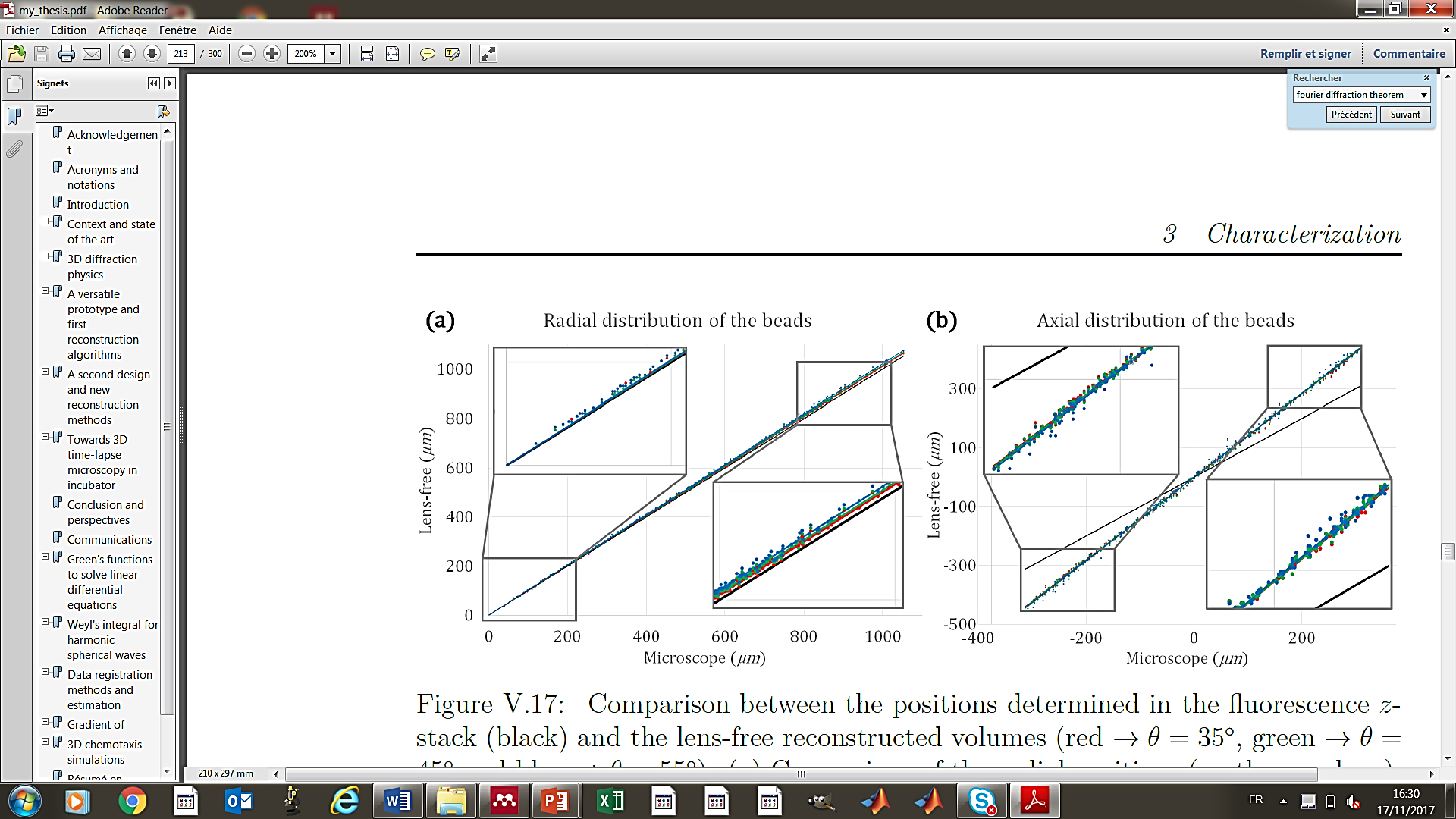


Figure S9. Comparison between the positions determined in the fluorescence z-stack (black) and the lens-free reconstructed volumes for $\theta=35^{\circ}$ (red), $\theta=45^{\circ}$ (green), $\theta=55^{\circ}$ (blue). (a) Comparison of the radial positions (on the $xy$-plane). (b) Comparison of the axial positions (along the z-axis).

| $n_{b}=409$ | Radial distributions | | | Axial distributions | | |
| --- | --- | --- | --- | --- | --- | --- |
|  | $\theta=35^{\circ}$ | $\theta=45^{\circ}$ | $\theta=55^{\circ}$ | $\theta=35^{\circ}$ | $\theta=45^{\circ}$ | $\theta=55^{\circ}$ |
| $s$ | 1.01 | 1.01 | 1.02 | 1.40 | 1.41 | 1.42 |
| $R^{2}$ | 0.9997 | 0.9997 | 0.9997 | 0.9991 | 0.9993 | 0.9991 |
| $\sigma(\mu m)$ | 3.7 | 3.5 | 3.9 | 6.6 | 6.4 | 7.4 |

Supplementary table 1. Results of the linear regression performed on the $n_{b}=409$ beads matched in the fluorescence z-stack and the three reconstructed volumes without the cap on the Petri dish. For $\theta\in\left\{ 35^{\circ}, 45^{\circ}, 55^{\circ} \right\}$, the slope $s$, the coefficient of determination $R^{2}$ and the standard deviation σ of the linear regressions are given both for the radial (on the $xy$-plane) and the axial (along the $z$-axis) distributions.

The results are presented in figure S9 and table 1. They are linear regressions $y=s.x$ which, compare the extracted positions in the reconstructed volume with their reference positions in the fluorescence z-stack which lie on the line $y=x$. On the $xy$-plane, to analyze the dilation, the distances $d^{b_{i}}=\left\| \vec{r}_{1,2}^{b_{i}} \right\|_{2}$are used rather than the $xy$-positions $x_{1,2}^{b_{i}}$ and $y_{1,2}^{b_{i}}$.

Both for the radial and axial distributions, the data lie precisely as expected on lines with coefficients of determination higher than $R^{2}$ > 0.999. For the radial distribution, the dilation effect noticed in figure S8.a is visible on the linear regressions and their slope $s$which increases with the angle as seen above. At the largest illumination angle ($\theta=55^{\circ},$and $s = 1.02$), this effect remains nevertheless under 2.5 % with a low standard deviation $\theta<4 \mu m$. The increase of the dilation factor with the angle $\theta$ is also observed on the z-axis. The standard deviation is worse than on the radial plane with values around $\sim7 \mu m$. Interestingly the value of the slopes $s = 1.41$ is similar for all tilting illumination angles $\theta$ but far from the ideal value of $s = 1$. Actually a scaling factor must be applied in the microscope acquisitions along the z-axis because of the refractive index mismatch between the sample in the water and the moving objective in the air. With $NA=0.13, n_{2}=n_{H2O}=1.33$ and $n_{1}=n_{air}$ air in equation, the scaling factor is given by:

$\frac{z_{H_{2}0}}{z_{air}}=\sqrt{\frac{n_{H_{2}0}^{2}-NA^{2}}{n_{air}^{2}-NA^{2}}}\simeq1.335$ (11)

which is close of the found slopes $s$.

*Resolution and xyz-sizing*

To get an estimation of the resolution and the sizing capabilities of the lens-free microscope and the associated reconstructions, a region of interest is selected and an isolated bead is chosen. The region of interest is a square of $256\times256$ pixels (${428}^{2} \mu m^{2}$) and the reconstructed volumes are composed of $256\times256\times256$ voxels of 1.673 $\mu m^{3}$. Figure S10 shows this region of interest as well as the beads by comparing the $xy$ and $xz$-average intensity projections of the fluorescence $z$-stack and the lens-free reconstruction without cap. On the $xy$-projections, isolated beads are perfectly identifiable and in the clusters of beads, the resolution appears sufficient to resolve the beads which lie in a same plane. For the rightmost cluster, the beads are agglutinated in the three directions and it becomes more difficult to separate the different beads. On the $xz$-projections it clearly appears that the lens-free reconstructions has a good sectioning capability. Nevertheless, the resolution is worse than on the $xy$-plane: the beads are elongated along $z$. The undersampling of the reconstruction, the illumination tilting angle $\theta\in\left\{ 35^{\circ}, 45^{\circ}, 55^{\circ} \right\},$ and the presence or not of the cap on the Petri dish, are parameters that can influence the quality of the reconstructions and the resulting resolution. Figures S11 and S12 compare the effects of these parameters. The cap on the Petri dish does not seem to influence the quality of the reconstruction. The voxel resolution 1.67 μm v.s. 3.34 μm does not influence the reconstruction resolution neither. Indeed, if the pixelization is visible on the low resolution projections, the full resolution projections do not provide better information. These projections are blurred and the edges of the beads are not sharper as it could be expected. Some tests (not presented here) showed that these projections are very similar with a scaling by a factor of 2 of the low resolution pictures with a bilinear interpolation. The voxel resolution has nevertheless an effect on the noise: the background in the low resolution reconstruction is darker and cleaner. An explanation for this effect is that no matter what is the voxel size, the pixel resolution in the dataset remains 1.67 μm. This means that there is redundant data in the low resolution reconstructions per voxel than for the full resolution reconstruction. This averages the noise in the data. The influence of the illumination tilting angle $\theta$ is much stronger. On the xy-views, the resolution slightly degrades as $\theta$ increases. This effect is particularly visible on the clusters where the resolution at $\theta=55^{\circ}$ is not sufficient anymore to dissociate the beads. But it is along the z-axis that this effect is the most visible: the elongation of the beads strongly deteriorates with the angle. To better quantify these effects, profiles are drawn along the x and the z-axis for the bead pointed in figures S10, S11 and S12. For the visualization in figures S13 and S14, the profiles are all normalized and centered. For each profiles, its full width at half maximum (FWHM$\sim2.35\sigma$) is measured along the z-axis. For the xy-plane, Gaussian distributions are fitted with parameters $x_{0}$(the translation of the curve), $\sigma$ (the standard deviation), $A$ (the amplitude) and $off$ (the offset):

$f\left( x \right)=A.e^{-\frac{{(x-x_{0})}^{2}}{2\sigma^{2}}}+off$ (12)


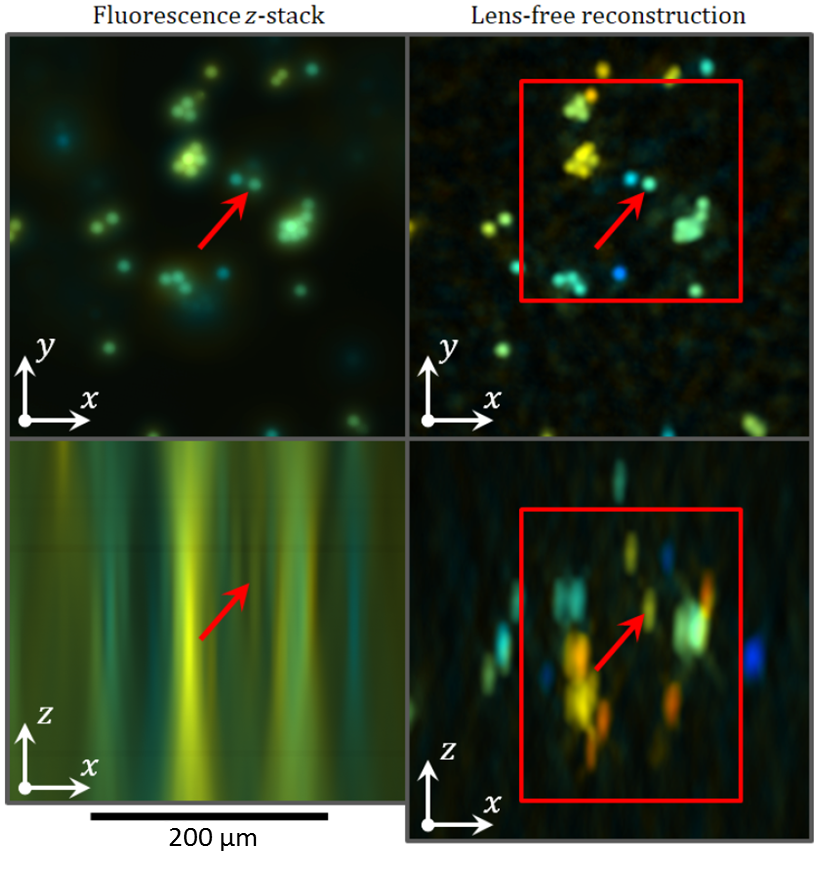


Figure S10. $xy$ and $xz$-average intensity projections of a region of interest of the fluorescence z-stack (left) and its counterpart in the lens-free reconstruction at full resolution (right, $\theta=35^{\circ}$, without cap). The red arrow points at the bead which is investigated. The red rectangles frame the regions which are compared in figures S12 and S13. On each view, the colors codes for the depth: the shallowest in blue, the deepest in red.


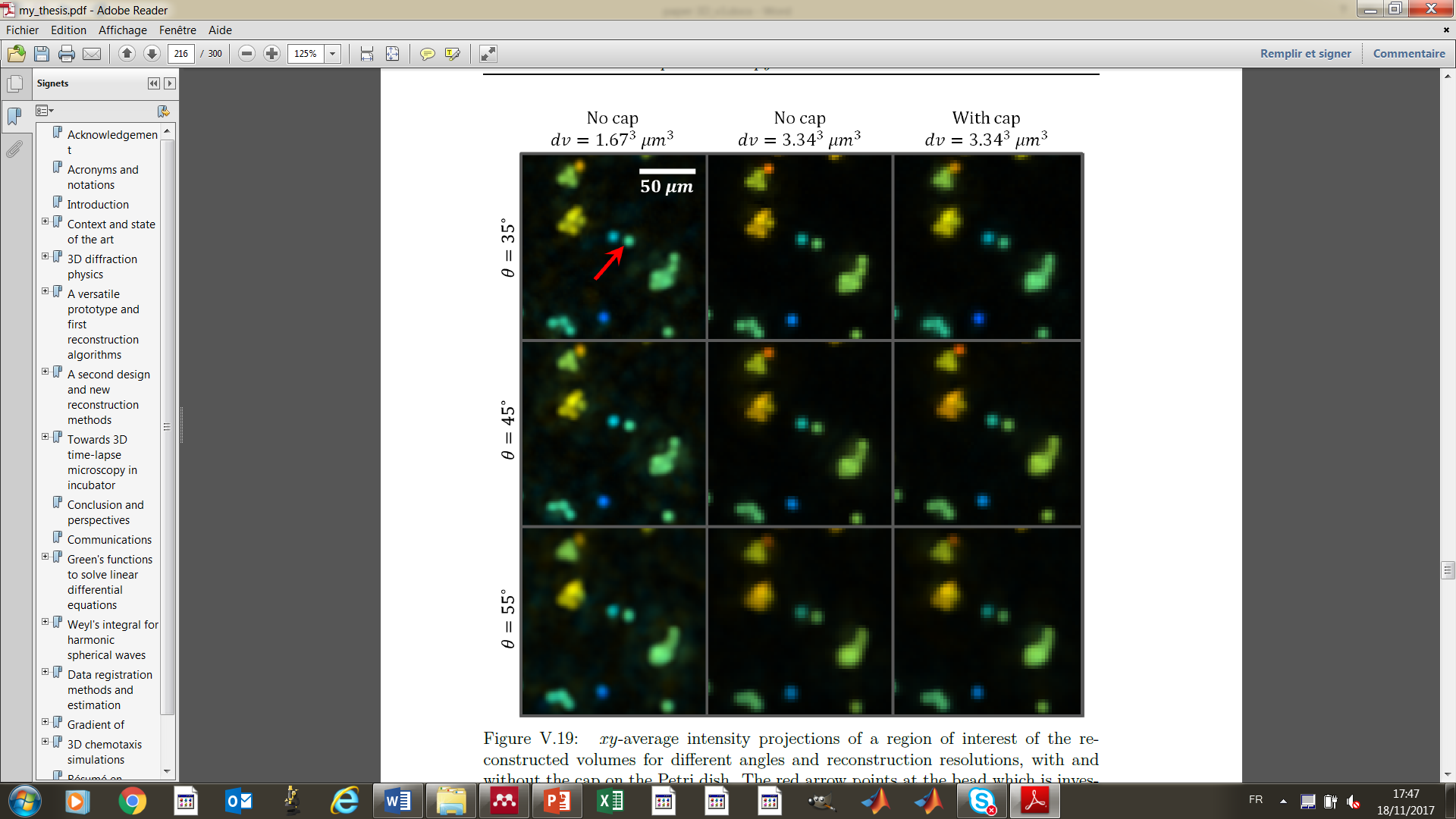


Figure S11. $xy$-average intensity projections of a region of interest of the reconstructed volumes for different angles and reconstruction resolutions, with and without the cap on the Petri dish. The red arrow points at the bead which is investigated. On each view, the colors codes for the depth: the shallowest in blue, the deepest in red.


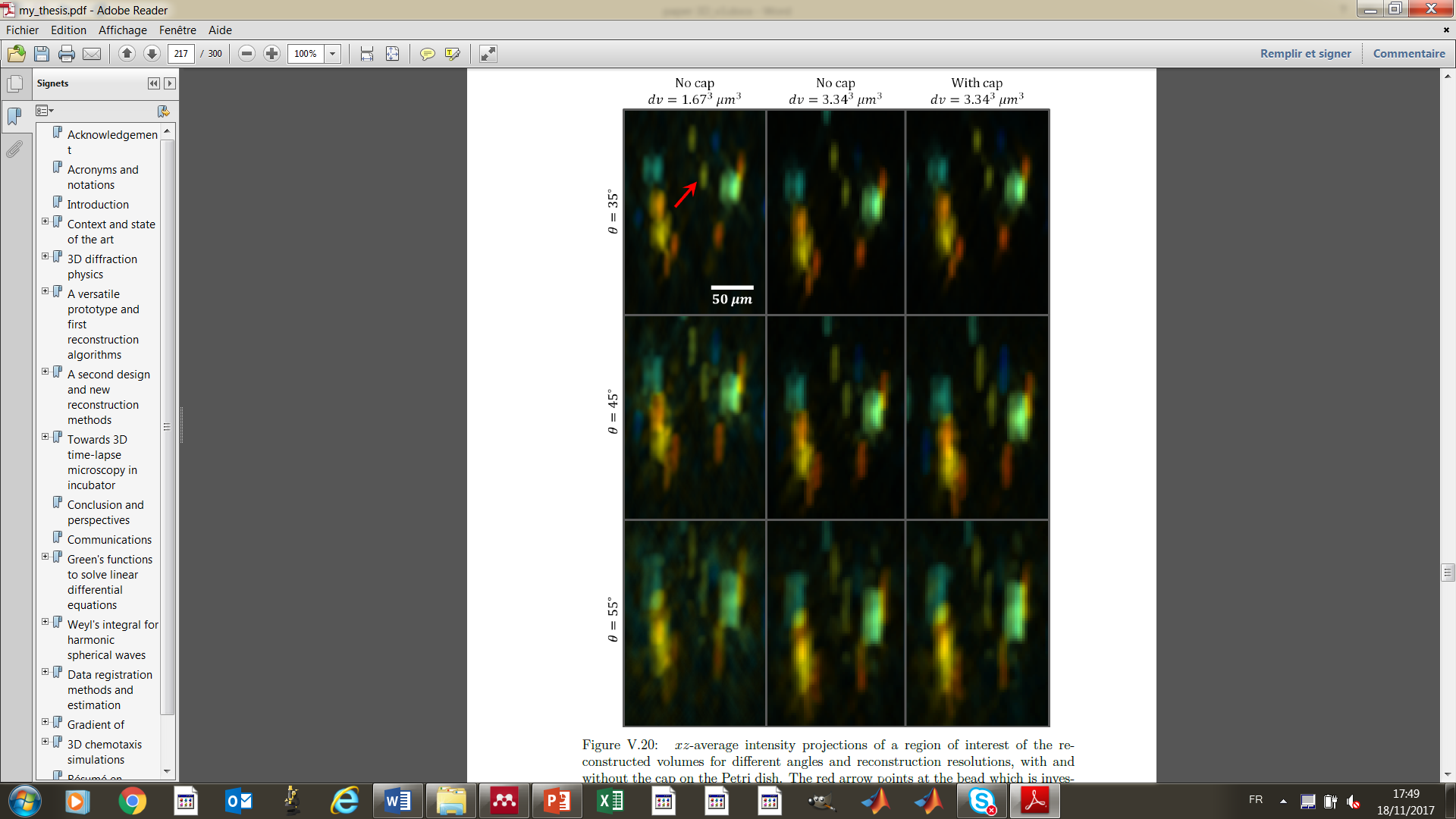


Figure S12. $xz$-average intensity projections of a region of interest of the reconstructed volumes for different angles and reconstruction resolutions, with and without the cap on the Petri dish. The red arrow points at the bead which is investigated. On each view, the colors codes for the depth: the shallowest in blue, the deepest in red.


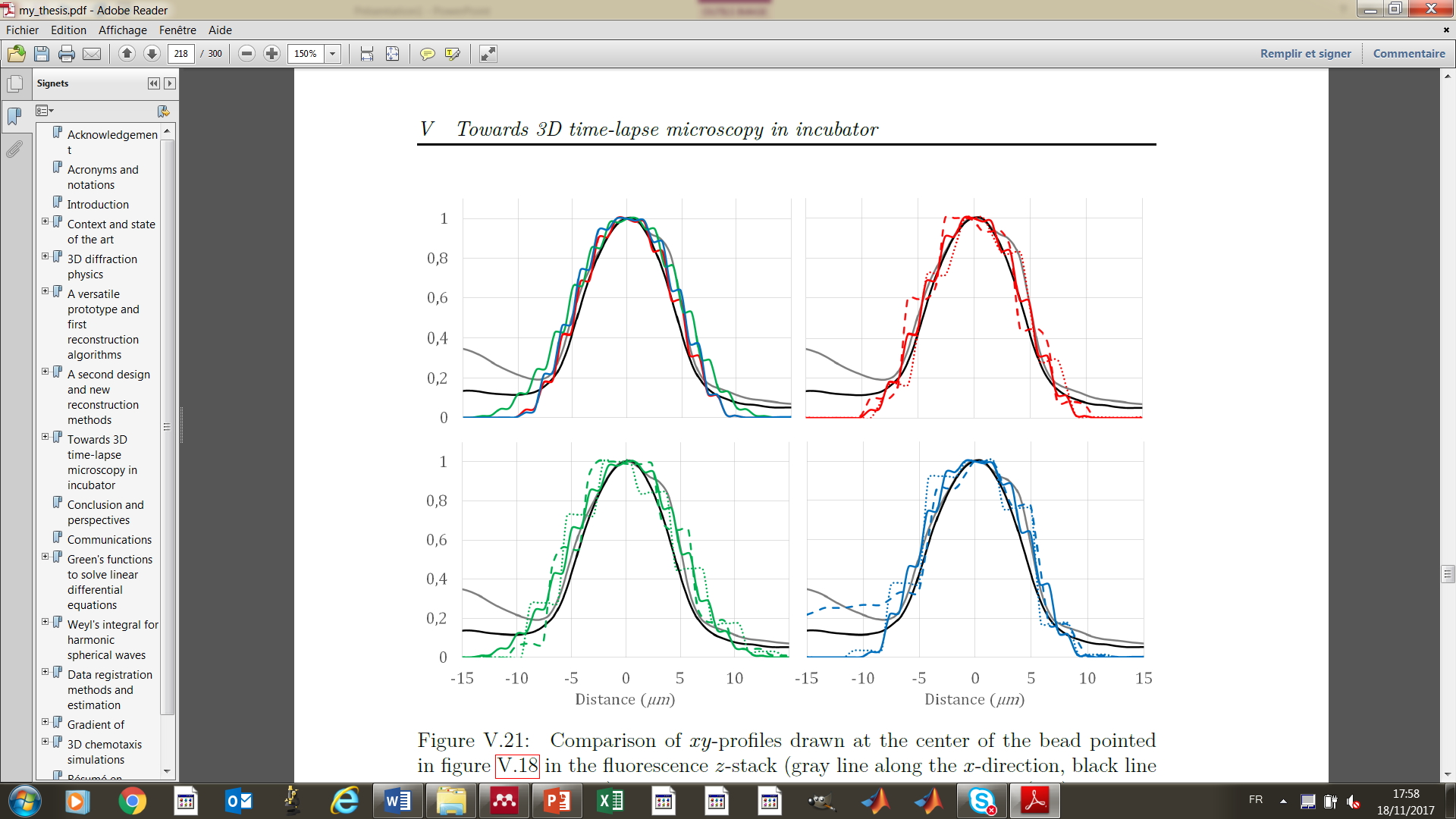


Figure S13. Comparison of xy-profiles drawn at the center of four different beads pointed in figure S10 in the fluorescence z-stack (gray line along the $x$-direction, black line along the y-direction) and the reconstructed volumes for $\theta=35^{\circ}$ (red), $\theta=45^{\circ}$ (green), $\theta=55^{\circ}$ (blue) at full resolution (solid line) and low resolution with (dotted line) and without (dashed line) the cap on the Petri dish.


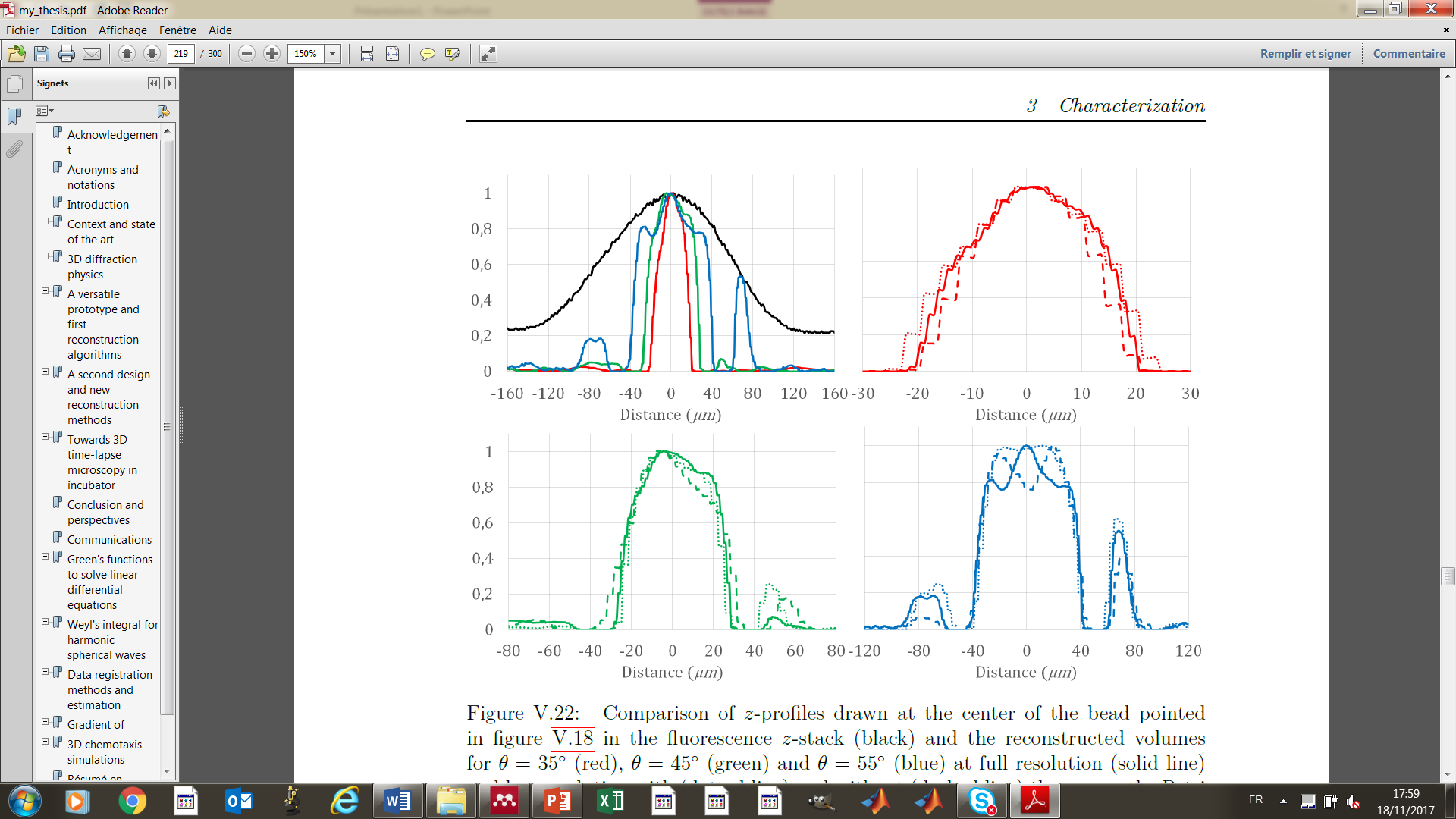


Figure S14. Comparison of z-profiles drawn at the center of the bead pointed in figure S10 in the fluorescence z-stack (black) and the reconstructed volumes for $\theta=35^{\circ}$ (red), $\theta=45^{\circ}$ (green), $\theta=55^{\circ}$ (blue) at full resolution (solid line) and low resolution with (dotted line) and without (dashed line) the cap on the Petri dish.

The results are presented in the table 2. As they are done on a single isolated bead these results cannot be used to quantify the resolution but they give an idea of the sizing capabilities of the system. On the radial plane, the curves present a similar FWHM around 9 to 10 μm which, is in a good agreement with the fluorescence measurements (figure S10). The presence or the absence of the cap on the Petri dish does not significantly change the measured FWHMs, the reconstruction conserves its quality. This means that the regularization is efficient to get rid of the structured background added by the presence of the cap in the signal. The FWHMs are not influenced by the value of the illumination tilting angle $\theta.$ This is in contradiction with the previous qualitative observation of a loss of resolution in the clusters (see figure S10). This may be due to the fact that this is an isolated bead on which the regularization is efficient compared to the clusters where a lack of resolution will be enhanced by the regularization which fosters an extended homogeneous object. The curves of figure S13 confirm the conclusions of the analysis of figure S11. The resolution of the reconstruction and the presence of the cap on the Petri dish do not have much influence, giving similar FWHMs. The angle illumination tilting angle $\theta$ has a strong influence on the sizing capabilities along the $z$-axis. All the lens-free reconstructions are better than the $z$-profile in the fluorescence $z$-stack. There is a strong disparity in the three cases with an elongation shifting from ~30 μm for $\theta$ =35° to ~70 μm for $\theta=55^{\circ}$. They are thus several times bigger than the 10 μm theoretical diameter of the beads. The sizing capabilities are consequently strongly diminished along the z-axis with an overestimation of at least ~20 μm. These FWHMs give the low limit of the axial resolution which cannot be expected to be better than these values.

|  | Fluo | Full resolution | | | No cap | | | With cap | | |
| --- | --- | --- | --- | --- | --- | --- | --- | --- | --- | --- |
| $\theta$ | 0° | 35° | 45° | 55° | 35° | 45° | 55° | 35° | 45° | 55° |
| $x (y)$ | 9.1 (8.3) | 9.1 | 10.5 | 9.5 | 9.0 | 11 | 9.7 | 9.2 | 10.0 | 10.6 |
| $z$ | 134 | 32 | 49 | 75 | 34 | 44 | 70 | 28 | 51 | 74 |

Supplementary table 2. Table of the measured full widths at half maximum in the different conditions. The values are given in microns.

**References**

1. Wolf, E. Three-dimensional structure determination of semi-transparent objects from holographic data. *Opt. Commun.* **1,** 153–156 (1969).

2. Sung, Y. *et al.* Optical diffraction tomography for high resolution live cell imaging. *Opt. Express* **17,** 266–277 (2009).

3. Liu, P. Y. *et al.* Cell refractive index for cell biology and disease diagnosis: past, present and future. *Lab Chip* **16,** 634–644 (2016).

4. Gerchberg, R. Holography without fringes in the electron microscope. *Nature* **240,** 404–406 (1972).

5. Medoff, B. P., Brody, W. R., Nassi, M. & Macovski, A. Iterative convolution backprojection algorithms for image reconstruction from limited data. *J. Opt. Soc. Am.* **73,** 1493 (1983).

6. Donoho, D. L. Compressed sensing. *IEEE Trans. Inf. Theory* **52,** 1289–1306 (2006).

7. Denis, L., Lorenz, D., Thiébaut, E., Fournier, C. & Trede, D. Inline hologram reconstruction with sparsity constraints. *Opt. Lett.* **34,** 3475–3477 (2009).

8. Rudin, L. I., Osher, S. & Fatemi, E. Nonlinear total variation based noise removal algorithms. *Phys. D Nonlinear Phenom.* **60,** 259–268 (1992).

9. Charbonnier, P., Blanc-F??raud, L., Aubert, G. & Barlaud, M. Deterministic edge-preserving regularization in computed imaging. *IEEE Trans. Image Process.* **6,** 298–311 (1997).

10. Nocedal, J. Updating quasi-Newton matrices with limited storage. *Math. Comput.* **35,** 773–773 (1980).

11. Momey, F. *et al.* Lensfree diffractive tomography for the imaging of 3D cell cultures. *Biomed. Opt. Express* **7,** 949 (2016).

12. Berdeu, A. *et al.* Comparative study of fully three-dimensional reconstruction algorithms for lens-free microscopy. *Appl. Opt.* **56,** 3939 (2017).
